# [Fe_2_L_3_]^4+^ Cylinders Derived from Bis(bidentate) 2-Pyridyl-1,2,3-triazole “Click” Ligands: Synthesis, Structures and Exploration of Biological Activity

**DOI:** 10.3390/molecules18066383

**Published:** 2013-05-29

**Authors:** Sreedhar K. Vellas, James E. M. Lewis, Madhu Shankar, Alia Sagatova, Joel D. A. Tyndall, Brian C. Monk, Christopher M. Fitchett, Lyall R. Hanton, James D. Crowley

**Affiliations:** 1 Department of Chemistry, University of Otago, P. O. Box 56, Dunedin 9054, New Zealand; 2 National School of Pharmacy, University of Otago, Dunedin 9054, New Zealand; 3 Sir John Walsh Research Institute, P.O. Box 647, Dunedin 9054, New Zealand; 4 Faculty of Dentistry, University of Otago, Dunedin 9054, New Zealand; 5 Department of Chemistry, College of Science, University of Canterbury, Christchurch 8140, New Zealand

**Keywords:** iron(II), CuAAC, metallosupramolecular cylinders, biological activity

## Abstract

A series of metallosupramolecular [Fe_2_L_3_](BF_4_)_4_ “click” cylinders have been synthesized in excellent yields (90%–95%) from [Fe(H_2_O)_6_](BF_4_)_2_ and bis(bidentate) pyridyl-1,2,3-triazole ligands. All complexes were characterized by elemental analysis, IR, UV-vis, ^1^H-, ^13^C- and DOSY-NMR spectroscopies and, in four cases, the structures confirmed by X-ray crystallography. Molecular modeling indicated that some of these “click” complexes were of similar size and shape to related biologically active pyridylimine-based iron(II) helicates and suggested that the “click” complexes may bind both duplex and triplex DNA. Cell-based agarose diffusion assays showed that the metallosupramolecular [Fe_2_L_3_](BF_4_)_4_ “click” cylinders display no antifungal activity against *S. cerevisiae.* This observed lack of antifungal activity appears to be due to the poor stability of the “click” complexes in DMSO and biological media.

## 1. Introduction

**Figure 1:**
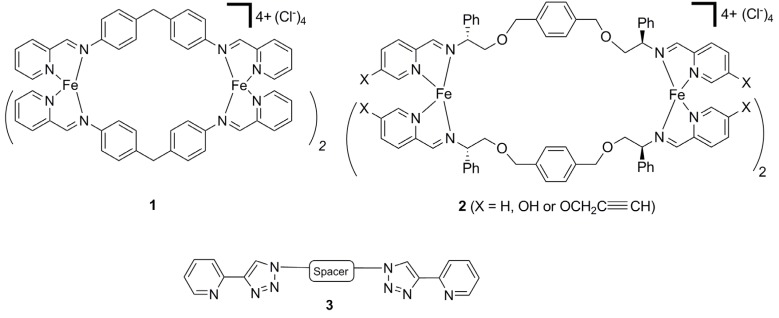
Hannon’s (compound **1**) and Scott’s (compound **2**) biologically active [Fe_2_L_3_]^4+^ helicates and the CuAAC “click”-derived bis(bidentate) ligands **3**.

Helicates [1,2,3,4,5,6] are one of the most studied types of discrete metallosupramolecular architectures. With judicious choice of the metal ion and linker ligand cyclic-, doubly-, triply- or quadruply-stranded helicates can be generated. These systems display interesting electronic [7,8,9], optical [10,11,12,13], catalytic [14] and molecular recognition [15,16,17,18,19,20] properties. They have also been used as building blocks to generate novel mechanically interlocked systems [21]. Pioneering work by Hannon and co-workers [22] showed that the triply stranded [Fe_2_L_3_]^4+^ helicate **1** (Figure 1) displays unique biological properties [23,24]. This tetracationic diiron(II) cylinder binds strongly and non-covalently to the major groove of duplex DNA [25,26,27] and, even more remarkably, can bind at the center of three-way (Y-shaped) DNA junctions [28,29]. The interaction of **1** with duplex DNA induces intramolecular DNA coiling [25,26,27]. Additionally, **1** has been shown to display both anti-cancer [30] and anti-bacterial [31] properties, but is not mutagenic or genotoxic [30]. Recently, Scott and co-workers reported that the related flexicates **2** (Figure 1) can bind DNA and act as anti-bacterial agents [32].

Due to its reliability, mild reaction conditions and wide substrate scope the copper(I)-catalyzed 1,3-cycloaddition of organic azides with terminal alkynes (the CuAAC “click” reaction) [33,34,35,36,37,38] has become the reaction of choice in the last decade for functional molecule synthesis in a wide range of fields, including the biological [39,40,41,42,43] and materials sciences [44,45,46,47,48]. This synthetic versatility has attracted the attention of coordination chemists and a vast array of 1,4-disubstituted-1,2,3-triazole- containing ligand architectures and the corresponding “click” complexes have been developed in the past five years [49,50,51,52]. We [53,54,55,56,57] and others [58,59] have explored the use of “click” ligands in the development of functionalized metallosupramolecular architectures, and have shown that metallo-macrocycles, cages and coordination polymers can be generated with these 1,4-disubstituted-1,2,3-triazole-containing ligands. In that work we developed a small family of bis-2-(1-*R*-1*H*-1,2,3-triazol-4-yl)pyridine ligands **3** (Figure 1) and showed that they assemble into metallomacrocyclic species with Ag(I) ions [57]. We reasoned that these ligands should react with Fe(II) ions to generate metallosupramolecular helicates of a similar size and shape to **1** and **2**. Given the wide substrate scope of the CuAAC “click” reaction used to construct the ligands, it should be possible to prepare a library of helicates that would enable convenient structure-activity studies. Herein we report the synthesis and biological properties [60,61,62,63,64,65,66,67,68,69] of a small family of Fe(II) “click” helicates. 

## 2. Results and Discussion

### 2.1. Synthesis of Model Iron(II) Complexes

**Scheme 1:**
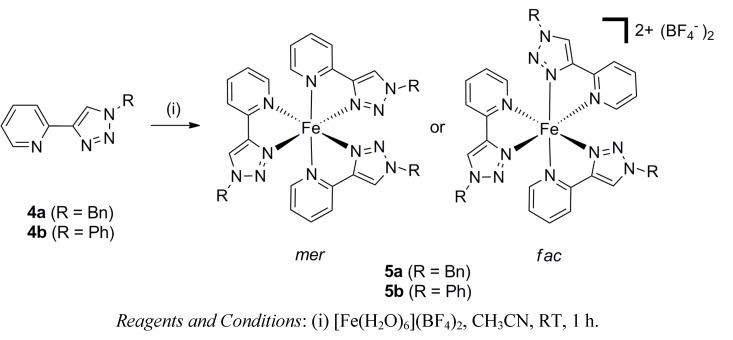
The synthesis of the model iron(II) complexes **5a**–**b**.

As tris(bidentate) Fe(II) complexes of the 2-(1-*R*-1*H*-1,2,3-triazol-4-yl)pyridine ligands **4a**–**b** [70,71] had not been described when we began this work, we initially examined the formation of the model Fe(II) complexes **5a**–**b** [72,73]. These complexes were prepared by reacting one of the ligands, either **4a** or **4b** (3 eq.), with iron(II) tetrafluoroborate hexahydrate (1 eq.) in acetonitrile at room temperature (Scheme 1). These compounds were isolated in excellent yields (90%–95%) as X-ray quality crystals by vapor diffusion of diethyl ether into the acetonitrile reaction mixtures. The presence of both the ligand (3000–2900 cm^−1^) and the BF_4_^−^ counter-anions (1049 cm^−1^) in the isolated orange red solids was confirmed by IR spectroscopy, and elemental analyses were consistent with the expected [Fe(**4a** or **4b**)_3_](BF_4_)_2_ formulation.

**Figure 2:**
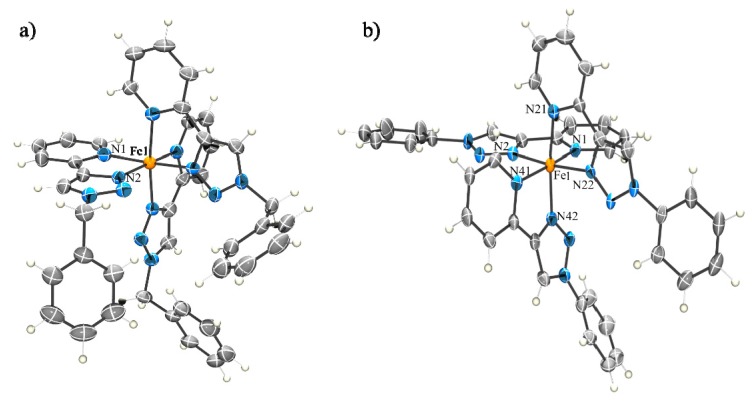
ORTEP diagrams of the solid state structures of *fac*-**5a** (a) and *mer*-**5b** (b). The thermal ellipsoids are shown at the 50% probability level. Solvent molecules and counteranions are omitted for clarity. Selected bond lengths (Å) and bond angles (°) for the complexes; **5a**: Fe1-N1 2.006(3), Fe1-N2 1.941(3) N1-Fe-N2 81.5(1); **5b**: Fe1-N1 1.990(5), Fe1-N21 2.007(4), Fe1-N41 1.987(4), Fe1-N2 1.918(4), Fe1-N22 1.947(4), Fe1-N42 1.940(4), N1-Fe-N2 80.6(2); N21-Fe-N22 80.8(2); N41-Fe-N42 81.7(2).

The molecular structures of the complexes **5a**–**b** were confirmed using X-ray crystallography (Figure 2 and Supporting Information). Both complexes were found to contain the expected [Fe(**L**)_3_]^2+^ cations with three of the 2-(1-*R*-1*H*-1,2,3-triazol-4-yl)pyridine ligands **4a**–**b** coordinated to the iron atoms in a pseudo-octahedral arrangement. The Fe-N_pyridine_ bond lengths vary between 1.987(4)–2.007(4) Å and Fe-N_triazole_ bond lengths ranges from 1.919(4)–1.948(4) Å. These data are consistent with the formation of the low-spin Fe(II) complexes. Interestingly, the complex **5a** crystallizes exclusively as the *fac* isomer, whereas **5b** is found to exist only as the *mer* isomer in the solid state. This diastereoselectivity is presumably caused by crystal packing effects. In contrast, the analogous ruthenium(II) complexes of these ligands exist as a 1:1 mixture of the *mer* and *fac* isomers in solution [74,75]. ^1^H-NMR spectroscopy was employed to further examine this behavior. 

**Figure 3:**
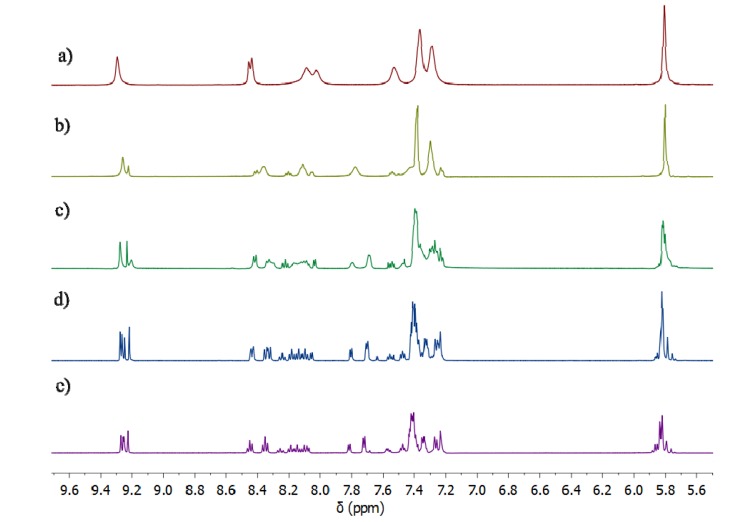
Partial ^1^H-NMR (500 MHz, *d*_6_-acetone) spectra of the Fe(II) model complex, **5a** at (**a**) 308 K, (**b**) 273 K, (**c**) 248 K, (**d**) 223 K, (**e**) 208 K.

^1^H-NMR spectra of the iron(II) model complexes **5a**–**b** recorded at room temperature in *d*_3_-acetonitrile and *d*_6_-acetone (Figure 3 and Supporting Information) show a simple pattern containing one set of proton signals, consistent with the quantitative formation of a single metal-containing species. Compared with the spectra of the ligands **4a**–**b**, the downfield shift of the proton signals of the corresponding iron complexes indicates metal complexation in solution (Figure 3 and Supporting Information). Closer inspection of the spectra shows that most of the peaks are broad at room temperature, indicative of some type of fluxional process. Variable-temperature (VT) ^1^H-NMR studies in *d*_6_-acetone were used to investigate the possible interconversion of the two diasteriomeric (*mer* and *fac*) forms of the model iron(II) complexes. Figure 3 shows the results for compound **5a**. At, and slightly above, room temperature (298 and 308 K) the spectra of **5a** display a single set of broadened resonances. As the solution is cooled these resonances sharpen and then split into two sets of peaks, consistent with the presence of both the *mer* and *fac* isomers of **5a** at around 208 K. These VT ^1^H-NMR data suggest that both the *mer* and *fac* isomers are present in solution, but at room temperature their interconversion on the NMR time scale leads to a single set of broad ^1^H-NMR resonances. This behaviour differs from that observed for structurally similar iron(II) [76] and analogous ruthenium(II) [74] complexes and suggests the Fe(II) complexes **5a**-**b** are stereochemically more labile than these related compounds. However, this lability is important, as access to the *fac* isomer is required for the bis(bidentate) ligands **3** to assemble into iron(II) metallosupramolecular cylinders.

### 2.2. Synthesis of Iron(II) Metallosupramolecular Cylinders

**Scheme 2:**
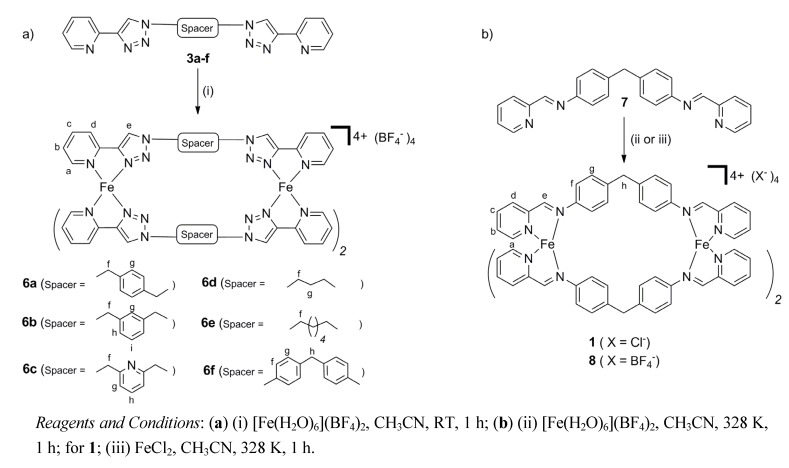
The synthesis of the iron(II) metallosupramolecular cylinders, **1**, **6a**–**f** and **8**.

The iron(II) metallosupramolecular cylinders **6a**–**f** were synthesized in an analogous fashion to the model complexes **5a**–**b**. The complexes were prepared by reacting each one of the ligands **3a**–**f** (3 eq.), with iron(II) tetrafluoroborate hexahydrate (2 eq.) in acetonitrile at room temperature (Scheme 2). The compounds were isolated in excellent yields (90%–95%) as either X-ray quality orange crystals (compounds **6a**–**d**, **6f**) or microcrystalline powders (compound **6e**) by vapor diffusion of diethyl ether into the acetonitrile reaction mixtures. Hannon’s related iron(II) metallosupramolecular cylinders **1** and **8** (X = BF_4_^−^) were also synthesized to allow direct comparison of the structural and biological properties of these complexes with **6a**–**f**. Complexes **1** and **8** were prepared by reacting the ligand **7** (3 eq.) either with iron(II) tetrafluoroborate hexahydrate (2 eq.) or iron(II) chloride, respectively, in acetonitrile at 328 K for one hour and isolated as purple solids in good yields (90%–94%, Scheme 2). All Fe(II) metallosupramolecular cylinders were characterized by elemental analysis, IR, UV-vis, ^1^H-, ^13^C- and DOSY-NMR spectroscopy and for **1** and **8**, by ESI-MS. The presence of both the ligand (3000–2900 cm^−1^) and the BF_4_^−^ counter-anions (1049 cm^−1^) in the isolated orange–red (compounds **6a**–**f**) or purple (compound **8**) solids was confirmed by IR spectroscopy and elemental analyses were consistent with the expected [Fe_2_(**L**)_3_](BF_4_)_4_ formulation.

#### 2.2.1. Solid State Structures

**Figure 4:**
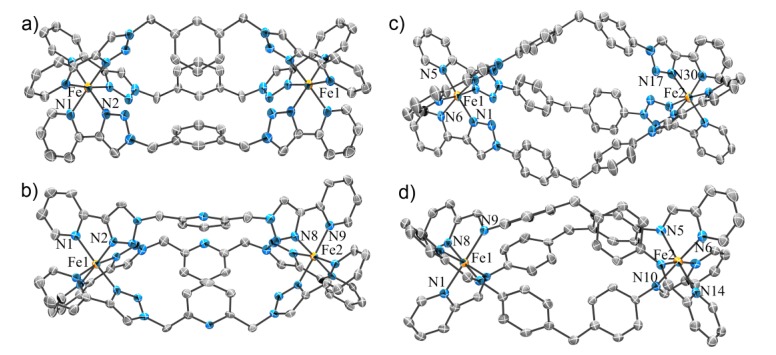
ORTEP diagrams of the solid state structures of the iron(II) metallosupramolecular cylinders, (a) **6b**, (b) **6c**, (c) **6f**, and (d) **8**. The thermal ellipsoids are shown at the 50% probability level. Solvent molecules, hydrogen atoms and counteranions are omitted for clarity. Selected bond lengths (Å) and bond angles (°) for the complexes; **6b**: Fe-N1 1.998 (3), Fe-N2 1.923(3); N1-Fe-N2 81.19(13); **6c**: Fe1-N1 2.001(4), Fe1-N2 1.944(3); N1-Fe-N2 81.05(15); **6f**: Fe1-N1 1.938, Fe1-N6 2.009, N1-Fe1-N6 81.30; **8:** Fe1-N1 1.994(4), Fe1-N3 2.010(4); N1- Fe- N3 80.65(15).

X-ray quality single crystals were isolated for the complexes **6a**–**d**, **f** and **8** but they all diffracted modestly and in certain cases synchrotron radiation was required to obtain satisfactory diffraction. Although the weak diffraction was, at least in part, due to the presence of multiple disordered solvent molecules within the crystal lattice (*vide infra*), the cationic cylinders were all readily identified. In all cases the crystals contained the expected iron(II) metallosupramolecular cylinders [Fe_2_L_3_]^4+^, along with some additional diffuse electron density peaks due to BF_4_^−^ counter-anions and solvent (usually CH_3_CN) molecules (Figure 4 and Supporting Information). The cationic cylinders were all well resolved and modeled completely. Where possible, BF_4_^−^ anions and solvent molecules were also modeled; the SQUEEZE routine within PLATON [77] was applied to the structural model before the final refinement due to the highly diffuse nature of some of the electron density associated with these species. Full details of the structural refinement and the crystallographic data are reported in the supporting information. Our X-ray data for the cylinders **6a** and **6d** showed these structures to be identical to those reported during the course of our study by Petitjean and co-workers [78]. 

Each of the pyridyl-1,2,3-triazole complexes **6a**–**d**, **f** contained the expected [Fe_2_L_3_]^4+^ cations and like the model complexes **5a**–**b**, the bond lengths and angles were consistent with the presence of low-spin iron(II). For the most part these complexes obey the odd-even rule [6,79,80]. Compounds **6b**–**d** [78], with an odd number of carbons in their spacer units, crystalize as the Λ∆-mesocate, whereas **6a** [78], with an even number of carbons in its spacer unit, was a racemic mixture of the ∆∆- and ΛΛ-helicates. The exception is **6f**, which despite having an odd number of atoms in its spacer, crystallizes as a racemic mixture of the ∆∆- and ΛΛ-helicates. This was not unexpected as the pyridyl imine complexes, **1** [81] and **8** (*vide infra*), with the analogous spacer unit, exist exclusively as helicates in both the solid and solution state. Ligand **3c** has two different potential chelate pockets ‒ the bidentate “regular” pocket and a central “inverse” tridentate pocket. Although tridentate complexes of similar “inverse click” ligands [82,83] are known, complex **6c** forms the desired [Fe_2_L_3_]^4+^ cylinder. This is presumably because coordination through the N3 nitrogen of the 1,2,3-triazole units, as observed for the bidentate coordination mode, is more thermodynamically favored [70,84]. Related Cu(II) complexes show similar behavior [85]. As the central pyridyl nitrogen atom of **6c** is not involved in any coordination interactions, complexes **6b** and **6c** are essentially isostructural (Supporting Information). Complex **6f** has a larger, more rigid spacer unit than the other cylinders and in the solid state this complex has an accessible central cavity filled with two acetonitrile “guest” molecules. None of the other cylinders have a cavity and the complexes with benzyl/pyridyl linkers display stabilizing π-π interactions between their central aromatic rings.

The solid state structure of **8** (the BF_4_^−^ analogue of Hannon’s complex **1**) was also determined using X-ray crystallography. The compound has been crystallographically characterized previously as the PF_6_^−^ salt [81]. The cylinder **8** crystallizes as a racemic mixture of the ∆∆- and ΛΛ-helicates. As expected, changing the counter-anion from PF_6_^−^ to BF_4_^−^ has little effect on the structure of the cylinder (Supporting Information). The structure of **8** allows a direct (size and shape) comparison with the “click” cylinders **6a**–**d**, **f**. The iron(II)-iron(II) distances (Table 1) and the overlays of the molecular structures (Supporting Information) for **8**, **6a**, **6b** and **6c** suggest that these complexes are structurally very similar. The same analysis for **8** with **6e** and **6f** showed that these pyridyl-triazole complexes are much smaller and significantly larger, respectively, than Hannon’s compounds. Their differing molecular size and shape might give rise to new biological properties.

**Table 1:** Metal - Metal distances within the Fe(II) cylinders.

Fe(II) cylinder	Fe(II)-Fe(II) distance
**1** (PF_6_^−^ salt) [81]	11.413(1) Å
**6a** [78]	11.391(3) Å
**6b**	12.182(3) Å
**6c**	12.234(1) Å
**6d** [78]	9.8777(7) Å
**6f**	14.579(4) Å
**8**	11.246(2) Å

#### 2.2.2. Solution Structures

**Figure 5:**
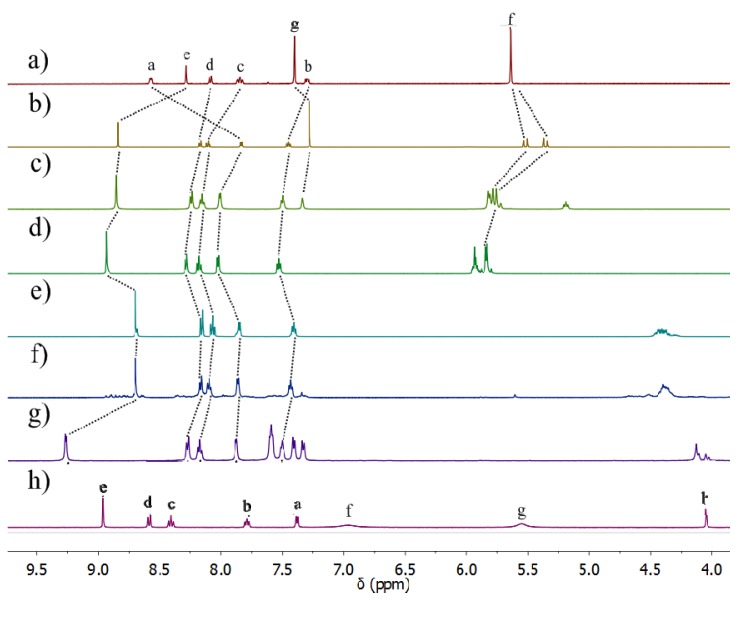
Partial ^1^H-NMR spectra (500 MHz, CD_3_CN, 298 K) of (a) ligand, **3a**, and the Fe(II) cylinders, (b) **6a** (c) **6b**, (d) **6c**, (e), **6d**, (f) **6e**, (g) **6f**, (h) **8**. The assignments correspond to the lettering shown in Scheme 2.

The solution structures of the cylinders **6a**–**f** and **8** were examined using ^1^H- and diffusion ordered (DOSY)-NMR spectroscopy (Figure 5, Table 2, and Supporting Information). The compounds **6a**–**d** and **8** appear to maintain their observed solid state structures in solution. ^1^H-NMR spectra (CD_3_CN, 298 K) of **6a**–**d** and **8** are generally sharp and display a set of peaks consistent with the quantitative formation of a single species in solution [Figures 5(a–h)]. ^1^H-DOSY-NMR spectroscopy of **6a**–**d** and **8** provides further support for this idea (*vide infra*). The triazole (compounds **6a**–**f**) or imine (compound **8**) proton signals in the ^1^H-NMR spectra of the cylinders are shifted downfield relative to their position in the “free” ligands (compounds **3a**–**f** and **7**), indicating complexation to Fe(II) ions. Consistent with the observed solid state structures the upfield shifts of proton resonances due to the aromatic spacer units of **6a**–**c**, **f** and **8** suggest the presence of π-π stacking interactions. The ^1^H-NMR spectra of **6e**–**f** were different. While they indicate that complexation had occurred, the spectrum of **6e** showed the formation of multiple species in solution, with a series of major peaks consistent with the formation of the [Fe_2_L_3_]^4+^ cylinder, along with smaller peaks attributed to the formation of larger oligomeric/polymeric species. This is probably due to the added flexibility of the hexyl linker, as similar behavior has been observed previously [6]. While the crystal structure of **6f** indicates exclusive formation of the helicate [Figure 4(c)], ^1^H-NMR analysis of the complex suggests that it exists as an approximately 1:1 mixture of the *rac*-helicate and the mesocate in solution [Figure 5(g)]. This is in contrast to the pyridyl imine complexes, **1** and **8**, which have the same spacer unit and are exclusively *rac*-helicates in solution and the solid state [Figure 4(d) and Figure 5(h)]. This difference in behavior appears related to the larger size of **6f** compared to **1** and **8**. In **1** and **8** the aryl rings of the spacer are in close contact and interdigitate, locking in the helical arrangement. The larger size of **6f** lessens this steric interdigitation of the spacer aryl groups making the mesocate arrangement more energetically accessible, thus giving rise to the mixture observed in solution [86].

^1^H-DOSY-NMR spectra (CD_3_CN, 298 K) were obtained for the ligands **3a**–**f** and **7** and the iron(II) cylinder complexes **6a**–**f** and **8** (Table 2, Supporting Information). All of the proton signals in the individual spectra of **6a**–**d** and **8** displayed the same diffusion coefficients (D) indicating that only one species was present in solution. The ligands (compounds **3a**–**f** and **7**) showed similar diffusion coefficients, ranging between 9.6–14.4 × 10^−1^^0^ m^2^·s^−1^, consistent with similar molecular sizes. The diffusion coefficients of the iron(II) cylinders (compounds **6a**–**f** and **8**) ranged between 3.0–6.9 × 10^−1^^0^ m^2^·s^−1^ indicating that all the cages are similar in size. The D_cylinder_/D_ligand_ ratios are approximately 0.3–0.5:1 suggesting that the iron(II) complexes are much larger than the free ligands, providing strong evidence for the retention of the cylinder architectures in CD_3_CN solution. The DOSY spectrum of **6e** was consistent with the existence of this complex as a mixture of the desired [Fe_2_L_3_]^4+^ cylinders, along with larger oligomeric/polymeric species. The major peaks in solution all displayed a diffusion coefficient of 4.9 × 10^−1^^0^ m^2^·s^−1^. This value is similar to the other [Fe_2_L_3_]^4+^ cylinders, indicating that this major species is indeed the dimetallic metallosupramolecular architecture. The other less intense peaks had smaller diffusion coefficients, suggesting the presence of larger oligomeric/polymeric systems in solution. The DOSY spectrum of **6f** showed two different sets of proton signals with the same diffusion coefficient of 3.0 × 10^−1^^0^ m^2^·s^−1^ indicating that two species present in solution are indeed the *rac*-(∆∆- and ΛΛ)-helicates and the (∆Λ)-mesocate.

ESI-MS spectra (CH_3_CN) were also obtained for the cylinders **6a**-**f** and **8**. Despite extensive efforts, including the use of pseudo cold-spray conditions [87,88], ions due to the intact [Fe_2_L_3_]^4+^ cylinders for the “click” complexes **6a**–**f** were not observed. Only peaks due to the “free” ligands and [FeL]^n+^ fragments could be detected for these complexes. In contrast, an isotopically resolved [Fe_2_L_3_]^4+^ ion was readily observed for the pyridyl imine complex **8** under standard ESI-MS conditions. The pyridylimine complexes appear much more stable than **6a**–**f** under the conditions of the ESI-MS experiment.

**Table 2:** Diffusion coefficients obtained from ^1^H DOSY spectra (CD_3_CN, 298 K).

Ligand	Diffusion coefficient (×10^−1^^0^ m^2^·s^−1^)	Fe(II) cylinder	Diffusion coefficient (×10^−1^^0^ m^2^·s^−1^)
**7**	12.9	**8**	6.9
**3a**	12.9	**6a**	4.7
**3b**	12.8	**6b**	4.7
**3c**	11.9	**6c**	4.0
**3d**	14.4	**6d**	3.9
**3e**	13.3	**6e**	4.9
**3f**	9.6	**6f**	3.0

#### 2.2.3. Electronic Spectra

**Figure 6:**
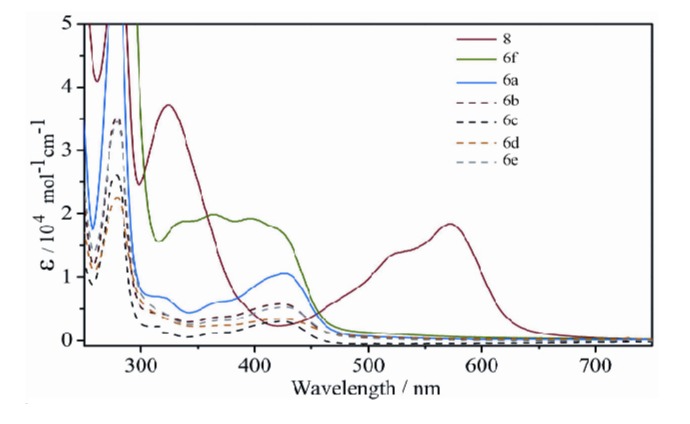
UV-vis. spectra of Fe(II) cylinders **6a**–**f** and **8** (10^−5^ M, CH_3_CN).

UV-vis spectroscopy (CH_3_CN) of compounds **5a**–**b**, **6a**–**f** and **8** was indicative of the formation of the iron(II) complexes in solution (Figure 6). Titration of [Fe(H_2_O)_6_](BF_4_)_2_ into acetonitrile solutions of all of the “click” ligands **3a**–**f** or **4a**–**b** gave rise to new absorption features in the visible region of the spectra (300–450 nm). All the complexes **5a**–**b**, **6a**–**f** display these absorption features with maxima for each compound observed at 420–430 nm (Figure 6 and Supporting Information). These absorption maxima are assigned as MLCT bands and are consistent with the formation of low spin *tris*-(diimine)Fe(II) complexes [22,32,76]. The MLCT bands of the “click” complexes **5a**–**b**, **6a**–**f** are blue-shifted relative to related the pyridylimine- **1**, **2** and **8** (λ_max_ = 570–600 nm) [22,32,76] and bipyridine (λ_max_ = 500–540 nm) [20,79]-based iron(II) complexes but occur in a similar region to complexes containing pyridylpyrazole chelators (λ_max_ = 423 nm) [89]. This shift to lower wavelength for the MLCT band of the “click” complexes compared to these other systems is consistent with the reduced π-acceptor nature of the triazole unit [74,75,89,90]. The combined solution data indicate that the [Fe_2_L_3_]^4+^ “click” cylinders are stable in acetonitrile solution and adopt similar structures to those observed in the solid state.

### 2.3. Molecular Modeling (Docking) and Biological Activity Studies

With the molecules in hand we examined the biological properties of the [Fe_2_L_3_]^4+^ cylinders, **6a**–**f** and **8**. Hannon and Scott have shown that a key biological target for these metallosupramolecular cylinders is DNA. We therefore examined the interaction between complexes **6a**–**f** and DNA *via* computational techniques. As the related [Fe_2_L_3_]^4+^ cylinders, **1** and **2**, have been shown to interact with both duplex and triplex DNA architectures, docking studies with both forms were carried out.

#### 2.3.1. Duplex Modeling

Molecular docking studies of metal complexes **1**(**8**), **6a**, **6b**, **6d**, and **6f** were carried out using generic B-DNA duplex structure (pdb3bse). The docking revealed that all Fe(II) cylinders, including **1**(**8**), fit into the major groove in a similar fashion to that reported previously by Erlend* et al.* [25] and Hannon* et al.* [27].

#### 2.3.2. Triplex Modeling

**Figure 7:**
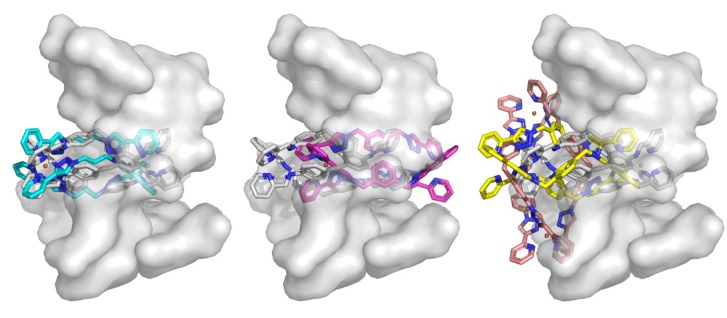
Docking of metal complexes into DNA triplex. The crystal structure of the triplex DNA (pdb2et0) is shown as a transparent surface representation with the Fe(II) complex, **1**(**8**), shown with white carbons. The smallest “click” complex, **6d**, best matches the crystal structure and is shown with cyan carbons (left). Complex **6b** is shown centrally located within the cavity (magenta carbons; center). The right panel shows the lowest ranking compounds **6a** and **6f** (yellow and brown respectively) adjacent to the cavity.

Molecular docking studies of metal complexes **6a**, **6b**, **6d** and **6f** were conducted using the triplex DNA structure (pdb2et0). Initially the Fe(II) complex **1**(**8**) was docked as a control. This procedure effectively replicated the reported crystal structure. Of the present complexes, **6d** ranked the highest, followed by **6b**, **6a** and **6f**. Complex **6d** binds in a fashion similar to **1**(**8**), with possible π interactions *via* the triazole ring, even though the ring is not planar with the DNA (Figure 7). Complex **6b** is shifted almost centrally within the cavity by approximately 6 Å. No clear π-stacking interactions are seen. Some close contacts exist between the non-aromatic spacer carbon and the thymine residue lining the cavity. Complex **6a** is shifted in the opposite direction (~4 Å; Figure 7). The largest “click” cylinder complex **6f** failed to dock within the central cavity of the DNA Y-junction. This complex binds on the surface of the DNA perpendicular to the other compounds. The docking studies support the postulate that some of the smaller “click” cylinders, **6b**–**d**, are of suitable size and shape to interact with both duplex and triplex DNA in a similar fashion to the known biologically active complexes, **1**(**8**) and **2**. These results led us to test the biological activity of the “click” cylinders using the yeast *S. cerevisiae* strain ADΔ/pABC3 [91].

#### 2.3.3. Biological Testing on Yeast Cells

**Figure 8:**
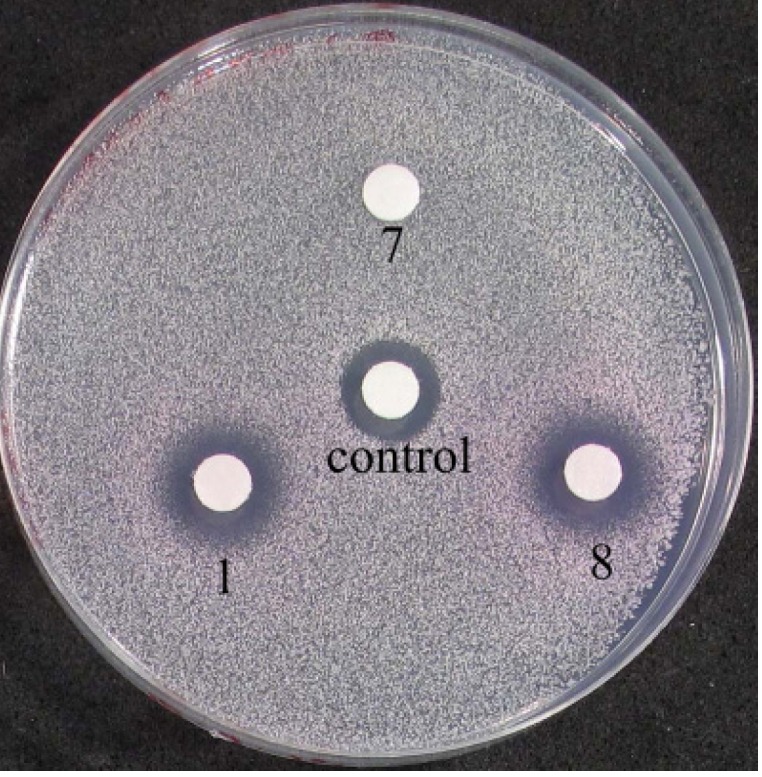
Agarose disc diffusion assay showing the effect of ligand, **7**, and Fe(II) complexes, **1** and **8** on the growth of *Saccharomyces cerevisiae* strain ADΔ/pABC3. Amphotericin B is used as a control.

Cell-based agarose diffusion assays were used to test the antifungal activity of both ligands, **3a**–**f**, **4a**–**b** and **7**, and the Fe(II) complexes, **1**, **5a**–**b**, **6a**–**f** and **8**. As the ligands and complexes were not soluble at high concentrations in aqueous media, stock solutions of each of the molecules were prepared in DMSO. Interestingly, none of the “click” ligands (compounds **3a**–**f** and **4a**–**b**) or complexes (compounds **5a**–**b** and **6a**–**f**) displayed any antifungal activity at pH 6.8 when 100 nmol was added per disk (Supporting Information). In contrast, the Fe(II) cylinders **1** and **8** displayed antifungal activity under identical conditions (100 nmol per disk). The cylinder **8** had a zone of inhibition of 12 mm and the related chloride salt **1** showed a zone of inhibition of 11 mm diameter (Figure 8). The control compound at 7 nmol per disk (amphotericin B) gave an inhibition zone of 9 mm. These results indicate that Hannon’s metallosupramolecular cylinders, **1** and **8** have modest antifungal activity compared with amphotericin B under the conditions of the experiment. These results suggest that suitably designed metallosupramolecular cylinders have potential as a novel class of antifungal agents. Despite the strong structural similarity between **1**(**8**) and **6a**–**f**, the lack of biological activity of the Fe(II) “click” cylinders may indicate that the molecular size and shape of the cationic cylinders are not the only factors important in the design of biologically active metallosupramolecular complexes.

#### 2.3.4. Solution Stability Studies of the Iron(II) Metallosupramolecular Architectures

Since the biological activities of the iron(II) metallosupramolecular architectures may depend on their stability, this property was tested for the Fe(II) cylinders **6a** and** 8** in the presence of a biologically relevant nucleophile (histidine) [92,93] and in DMSO solution using ^1^H-NMR and UV-vis spectroscopy and ESI-MS. 

**Figure 9:**
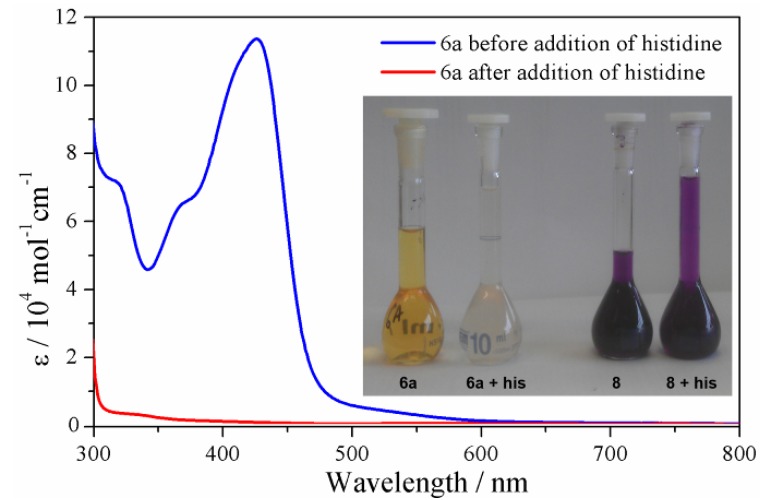
UV-vis spectra of the cylinder **6a** and a mixture of **6a** and histidine (6 eq.) in CD_3_CN/D_2_O (97:3). Inset photographs of **6a**, **6a** + histidine (6 eq.), **8**, and **8** + histidine (6 eq.) in CD_3_CN/D_2_O (97:3).

Competition experiments were carried out in CD_3_CN/D_2_O (97:3) at 40 °C using histidine as a surrogate for other biological nucleophiles. Both the Fe(II) cylinders, **6a** and **8**, were stable in the solvent mixture for a period of 24 h as judged by ^1^H-NMR spectroscopy and ESI-MS (for **8**). Addition of D,L-histidine monohydrochloride monohydrate (6 eq.) to a CD_3_CN/D_2_O (97:3) solution of the “click” cylinder **6a** led to instantaneous decomposition of the complex as shown by ^1^H-NMR and UV-vis spectroscopy (Supporting Information and Figure 9). The color of the solution changed immediately from orange/yellow to colorless and the MLCT (λ_max_ = 423 nm) in the UV-vis spectrum was completely extinguished, indicating that the cylinder was destroyed (Figure 9). This was confirmed by ^1^H-NMR spectroscopy as the **6a**/D,L-histidine containing mixture displayed only peaks due to the “free” ligand **3a**. The ESI-MS (positive mode) of this mixture also only displayed peaks due to the ligand **3a**. Presumably the iron(II) ions form neutral [Fe(his)_2_] [94] complexes which are not detected by ESI-MS under these conditions. Carrying out the same experiment with the pyridyl imine cylinder **8** gave a different result. The CD_3_CN/D_2_O (97:3) solutions of **8** were deep purple in color and lightened only slightly upon addition of D,L-histidine monohydrochloride monohydrate (Figure 9 inset, Supporting Information). The intense purple color remained even after 24 h. ESI-MS of the reaction mixture after 24 h showed the presence of a mixture of compounds, including free ligand, **7**, and the ligand precursors and the aldehyde precursor to the ligand. Isotopically resolved peaks due to the intact cylinder complex were also present. ^1^H-NMR spectroscopy of the mixture of **8** and histidine showed the presence of additional species in solution which corresponded to the free ligand, **7**, and the aldehyde precursor to the ligand. However, there were also peaks consistent with the intact cylinder complex and these remain even after 24 h.

The stability of the iron(II) metallosupramolecular cylinders in DMSO solution was examined using the same techniques. While acetonitrile solutions of **6a** were orange/yellow in color, dissolution of **6a** in DMSO gave a colorless solution that had no MLCT band (Supporting Information). ^1^H-NMR spectroscopy of **6a** in *d*_6_-DMSO was consistent with the UV-vis spectroscopy data. The position of the single set of broad resonances indicated that the Fe(II) cylinder had decomposed in the coordinating DMSO solvent (Supporting Information). Analogous experiments with **8** in DMSO indicated that pyridyl imine complexes were also somewhat unstable in the coordinating solvent. However, when **8** was dissolved in DMSO solution the deep purple color of the complex persists, and the intensity of the MLCT band is reduced. This indicates that the amount of the intact complex in DMSO was reduced compared to acetonitrile but some of the Fe(II) cylinder remains present in solution. 

These results suggest that the pyridylimine helicates, **1** and **8**, are far more stable than the “click” cylinders **6a**–**f** under the conditions used to carry out the biological assays. This difference explains why the pyridyl imine cylinders display antifungal activity, whereas the “click” cylinders do not. The high lability of these Fe(II) “click” complexes is due to two factors: 1) the weaker σ-donor ability of the ligands’ 1,2,3-triazole units, and 2) lone pair-lone pair repulsion between the uncoordinated N2 nitrogen atoms of the “click” ligands when they are in the *fac*-arrangement found in the cylinder structures. The weaker donor ability of the 2-(1-*R*-1*H*-1,2,3-triazol-4-yl)pyridine ligands compared to other diimines has been observed experimentally [95] and presumably results from the presence of the extra electronegative nitrogens in the 1,2,3-triazole units of the ligand. The enforced *fac*-arrangement of the 2-(1-*R*-1*H*-1,2,3-triazol-4-yl)pyridine chelate units may additionally destabilize the “click” cylinders relative to the pyridyl imine,* i.e.* the lone pair-lone pair repulsion between the uncoordinated N2 nitrogen atoms of the “click” ligands when they are in the *fac*-arrangement are unlikely to be favorable and further weaken the metal ligand interaction in comparison with systems that do not contain this motif.

## 3. Experimental

### 3.1. General Information

Unless otherwise stated, all reagents were purchased from commercial sources and used without further purification. Solvents were laboratory reagent grade with the following exceptions: dry acetonitrile was obtained by passing the solvent through an activated alumina column on a PureSolv TM solvent purification system (Innovative Technologies, Inc., Amesbury, MA, USA). ^1^H and ^13^C-NMR spectra were recorded on either a 400 MHz Varian 400 MR or Varian 500 MHz VNMRS spectrometers. Chemical shifts are reported in parts per million and referenced to residual solvent peaks (CD_3_CN: ^1^H δ 1.94, ^13^C δ 1.32, 118.26 ppm, *d*_6_-DMSO: ^1^H δ 2.50 ppm; ^13^C δ 39.52 ppm). Coupling constants (*J*) are reported in Hertz (Hz). Standard abbreviations indicating multiplicity were used as follows: m = multiplet, q = quartet, t = triplet, dt = double triplet, d = doublet, dd = double doublet, s = singlet. IR spectra were recorded on a Bruker ALPHA FT-IR spectrometer with an attached ALPHA-P measurement module. Microanalyses were performed at the Campbell Microanalytical Laboratory at the University of Otago. Electrospray mass spectra (ESI-MS) were collected on a Bruker micro-TOF-Q spectrometer. UV-visible absorption spectra were acquired with a Perkin Elmer Lambda-950 spectrophotometer in acetonitrile (10^−5^ M concentrations). The ligands **3a**–**f**, [57] **4a**–**b**, [70,71] and 7 [22] and the complex **1** [22] were prepared according to the literature procedures. 

### 3.2. General Method for the Synthesis of the Fe(II) Model Complexes ***5a***–***b***

An acetonitrile solution (3 mL) of iron(II) tetrafluoroborate hexahydrate (0.048 g, 0.14 mmol, 1 eq.) was added to an acetonitrile solution (3 mL) of one of the ligands **4a**–**b** (0.100 g, 0.43 mmol, 3 eq.). The resulting orange solution was stirred for one hour, filtered through cotton wool and vapor diffused with diethyl ether resulting in light red colored X-ray quality crystals of **5a** or **5b**. The crystals were isolated by filtration, washed with Et_2_O and dried *in vacuo*.

**5a**. Red crystals (0.110 g, 90%). MP: 140–142 °C. ^1^H-NMR (500 MHz, CD_3_CN): δ 8.78 (s, 3H, H_e_), 8.19 (d, *J* = 7.9 Hz, 3H, H_d_), 7.99–7.96 (m, 3H, H_c_), 7.78–7.76 (m, 3H, H_a_), 7.43–7.28 (m, 12H, H_b_, H_g_, H_i_), 7.21–7.16 (m, 6H, H_h_), 5.63 (s, 6H, H_f_). ^13^C-NMR (125 MHz, CD_3_CN): δ 155.9, 152.1, 150.8, 139.8, 134.1, 130.3, 129.1, 128.1, 127.3, 126.4, 123.7, 56.9. ATR-IR: υ (cm^−1^) 3123, 1645, 1623, 1584, 1497, 1452, 1428, 1356, 1328, 1277, 1245, 1216, 1156, 1126, 1049. Anal. calcd. for C_42_H_36_B_2_F_8_FeN_12_•(0.5H_2_O): C 53.67, H 4.19, N 17.47%; found: C 53.30, H 4.23, N 17.67%. UV-Vis (CH_3_CN) λ_max_/nm (ε/L mol^−1^cm^−1^) = 241 (4.4 × 10^4^), 280 (5.2 × 10^4^), 425 (9.5 × 10^3^).

**5b**. Red crystals (0.111 g, 91%). MP: 91–92 °C. ^1^H-NMR (500 MHz, CD_3_CN): δ 9.32 (s, 3H, H_e_), 8.27 (d, *J* = 7.8 Hz, 3H, H_d_), 8.12 (t, *J* = 7.8 Hz, 3H, H_c_), 7.86–7.83 (m, 3H, H_a_), 7.75 (d, *J* = 7.6 Hz, 6H, H_f_), 7.63–7.56 (m, *J* = 8.3 Hz, 9H, H_g_ & H_h_), 7.44–7.41 (m, 3H, H_b_). ^13^C-NMR (125 MHz, CD_3_CN): δ 156.3, 153.3, 151.6, 140.2, 137.4, 131.3, 131.1, 126.8, 124.4, 123.4, 121.7. ATR-IR: υ (cm^−1^) 3548, 3129, 1619, 1595, 1502, 1464, 1419, 1359, 1281, 1260, 1212, 1164, 1142, 1049, 1031. Anal. calcd. for C_39_H_30_B_2_F_8_FeN_12_•(0.5H_2_O): C 51.75, H 3.45, N 18.57%; found: C 52.00, H 3.60, N 18.12%. UV-Vis (CH_3_CN) λ_max_/nm (ε/L mol^−1^cm^−1^) = 240 (7.4 × 10^4^), 280 (9.0 × 10^4^), 425 (1.8 × 10^4^).

### 3.3. General Method for the Synthesis of the Fe(II) Cylinders ***6a***–***f***

An acetonitrile solution (3 mL) of iron(II) tetrafluoroborate hexahydrate (0.048 g, 0.14 mmol, 2 eq.) was added to an acetonitrile solution (3 mL) of one of the ligands **3a**–**f** (0.21 mmol, 3 eq.). The resulting orange solution was stirred at room temperature for 1 h then filtered through cotton wool and vapor diffused with diethyl ether resulting in orange/red X-ray quality crystals. The crystals were isolated by filtration and washed with Et_2_O and then vacuum dried. 

**6a**. Orange/red crystals (0.110 g, 94%). MP: > 230 °C. ^1^H-NMR (500 MHz, CD_3_CN): δ 8.83 (s, 6H, H_e_), 8.20–8.06 (m, 12H, H_d_ and H_c_), 7.83 (dt, *J* = 5.6, 1.2 Hz, 6H, H_a_), 7.44 (ddd, *J* = 7.4, 5.6, 1.5 Hz, 6H, H_b_), 7.27 (s, 12H, H_g_), 5.53 (s, 6H, H_f_), 5.33 (s, 6H, H_f_). ^13^C-NMR (125 MHz, CD_3_CN): δ 155.9, 153.9, 151.1, 140.1, 136.8, 129.5, 126.9, 125.6, 123.3, 55.9. ATR-IR: υ (cm^−1^) 3417, 3298, 3130, 1622, 1583, 1518, 1450, 1426, 1360, 1329, 1275, 1246, 1214, 1159, 1126, 1049, 1031. Anal. calcd for C_66_H_54_B_4_F_16_Fe_2_N_24_•(15H_2_O): C 41.45, H 4.43, N 17.58%; found: C 41.20, H 4.33, N 17.71%. UV/Vis (CH_3_CN) λ_max_/nm (ε/L mol^−1^cm^−1^) = 240 (2.6 × 10^4^), 279 (5.2 × 10^4^), 425 (1.0 × 10^4^).

**6b**. Orange/red crystals (0.110 g, 94%). MP >230 °C. ^1^H-NMR (500 MHz, CD_3_CN): δ 8.85 (s, 6H, H_e_), 8.24 (d, *J* = 7.9 Hz, 6H, H_d_), 8.16 (td, *J* = 7.8, 1.4 Hz, 6H, H_c_), 8.01 (d, *J* = 5.7 Hz, 6H, H_a_), 7.50 (t, *J* = 6.7 Hz, 6H, H_b_), 7.33 (s, 3H, H_g_), 5.84–5.81 (m, 6H, H_h_), 5.78 (s, 6H, H_f_), 5.75 (s, 6H, H_f_), 5.20 (t, *J* = 7.9 Hz, 3H, H_i_). ^13^C-NMR (125 MHz, CD_3_CN): δ 156.0, 154.0, 150.7, 140.1, 136.9, 130.6, 127.3, 126.8, 123.4, 123.3, 123.1, 54.8. ATR-IR: υ (cm^−1^) 3114, 1623, 1584, 1499, 1471, 1451, 1361, 1331, 1280, 1245, 1229, 1165, 1129, 1050. Anal. calcd for C_66_H_54_B_4_F_16_Fe_2_N_24_•(3H_2_O): C 46.73, H 3.57, N 19.82%; found: C 46.89, H 3.63, N 19.66%. UV/Vis (CH_3_CN) λ_max_/nm (ε/L mol^−1^cm^−1^) = 239 (3.0 × 10^4^), 277 (3.5 × 10^4^), 418 (5.6 × 10^3^).

**6c**. Orange/red crystals (0.110 g, 90%). MP: >230 °C. ^1^H-NMR (500 MHz, CD_3_CN): δ 8.89 (s, 6H, H_e_), 8.27 (d, *J* = 7.7 Hz, 6H, H_d_), 8.18 (td, *J* = 7.8, 1.4 Hz, 6H, H_c_), 8.02 (d, *J* = 5.5 Hz, 6H, H_a_), 7.54–7.49 (m, 6H, H_b_), 5.95–5.90 (m, 9H, H_g_ and H_h_), 5.81 (s, 12H, H_f_). ^13^C-NMR (125 MHz, CD_3_CN): δ 155.6, 155.4, 153.1, 150.4, 139.5, 138.2, 126.7, 126.3, 122.7, 55.8. ATR-IR: υ (cm^−1^) 3128, 1621, 1601, 1576, 1467, 1451, 1360, 1346, 1323, 1282, 1247, 1228, 1162, 1128, 1049. Anal. calcd for C_63_H_51_B_4_F_16_Fe_2_N_27_•(6H_2_O): C 43.16, H 3.62, N 21.57%; found: C 43.45, H 3.26, N 21.57%. UV/Vis (CH_3_CN) λ_max_/nm (ε/L mol^−1^cm^−1^): 239 (1.7 × 10^4^), 278 (2.6 × 10^4^), 424 (3.5 × 10^3^).

**6d**. Orange/red crystals (0.105 g, 90%). MP: >230 °C. ^1^H-NMR (500 MHz, CD_3_CN): δ 8.71 (s, 6H, H_e_), 8.18 (dd, *J* = 7.9, 1.1 Hz, 6H, H_d_), 8.08 (td, *J* = 7.8, 1.4 Hz, 6H, H_c_), 7.87 (d, *J* = 5.6 Hz, 6H, H_a_), 7.43 (td, *J* = 5.8, 2.9 Hz, 6H, H_b_), 4.46–4.34 (m, 12H, H_f_), 2.53–2.35 (m, 6H, H_g_). ^13^C-NMR (125 MHz, CD_3_CN): δ 155.9, 153.1, 149.9, 139.1, 126.7, 125.1, 122.1, 49.1, 31.1. ATR-IR: υ (cm^‒1^) 3132, 1621, 1581, 1563, 1472, 1454, 1438, 1393, 1361, 1336, 1279, 1248, 1204, 1166, 1129, 1102, 1051, 1029, 1015. Anal. calcd for C_51_H_48_B_4_F_16_Fe_2_N_24_•(6H_2_O): C 39.16, H 3.87, N 21.4%; found: C 38.92, H 3.54, N 21.39%. UV/Vis (CH_3_CN) λ_max_/nm (ε/L mol^−1^cm^−1^) = 238 (3.6 × 10^4^), 278 (2.2 × 10^4^), 425 (4.0 × 10^3^).

**6e**. Orange/red crystals (0.097 g, 90%). MP: >230 °C. ^1^H-NMR (500 MHz, CD_3_CN): chemical shift values for the major component δ 8.71 (s, 6H, H_e_), 8.15 (d, *J* = 8.0 Hz, 6H, H_d_), 8.08 (t, *J* = 7.8 Hz, 6H, H_c_), 7.86 (d, *J* = 5.6 Hz, 6H, H_a_), 7.44 (t, *J* = 6.7 Hz, 6H, H_b_), 4.42–4.33 (m, 12H, H_f_), 1.90–1.68 (m, 12H, H_h_), 1.20–1.14(m, 12H, H_g_). ^13^C-NMR (125 MHz, CD_3_CN): δ 155.1, 154.1, 150.5, 139.1, 126.7, 125.6, 122.1, 53.0, 30.9, 26.6. ATR-IR: υ (cm^−1^) 3129, 2930, 2860, 1619, 1580, 1455, 1432, 1360, 1279, 1214, 1161, 1049. Anal. calcd for C_60_H_66_B_4_F_16_Fe_2_N_24_•(5.5H_2_O): C 42.86, H 4.62, N 19.99%; found: C 47.73, H 4.31, N 19.61%. UV/Vis (CH_3_CN) λ_max_/nm (ε/L mol^−1^cm^−1^) = 240 (3.5 × 10^4^), 278 (3.5 × 10^4^), 420 (5.4 × 10^3^).

**6f**. Red crystals (0.115 g, 90%). MP: >230 °C. ^1^H-NMR (500 MHz, CD_3_CN): δ 9.26 (s, 6H, H_e_), 8.27 (d, *J* = 7.9 Hz, 6H, H_d_), 8.18 (t, *J* = 7.8 Hz, 6H, H_c_), 7.86 (d, *J* = 5.7 Hz, 6H, H_a_), 7.58 (dd, *J* = 8.1, 5.2 Hz, 12H, H_f_), 7.50 (t, *J* = 6.8 Hz, 6H, H_b_), 7.40 (d, *J* = 8.1 Hz, 6H, H_g_), 7.33 (d, *J* = 8.1 Hz, 6H, H_g1_), 4.13–4.05 (m, 6H, H_h_). ^13^C-NMR (125 MHz, CD_3_CN): δ 155.9, 153.6, 151.2, 144.1, 143.9, 140.3, 135.4, 131.2, 131.1, 127.1, 123.4, 123.2, 121.3. ATR-IR: υ (cm^−1^) 3363, 3127, 1620, 1579, 1515, 1464, 1414, 1359, 1279, 1259, 1216, 1164, 1050. Anal. calcd for C_81_H_60_B_4_F_16_Fe_2_N_24_•(5H_2_O + 3.5CH_3_CN): C 51.25, H 3.93, N 18.68%; found: C 51.24, H 3.79, N 19.01%. UV/Vis (CH_3_CN) λ_max_/nm (ε/L mol^−1^cm^−1^) = 245 (3.0 × 10^4^), 282 (5.3 × 10^4^), 410 (1.9 × 10^4^).

### 3.4. Synthesis of the Fe(II) Pyridylimine Cylinder ***8***

An acetonitrile solution (3 mL) of iron(II) tetrafluoroborate hexahydrate (0.026 g, 0.18 mmol, 2 eq.) was added to an acetonitrile solution (3 mL) of the ligand **7** (0.100 g, 0.27 mmol, 3 eq.) and heated at 55 °C for one hour which resulted in the formation of a purple colored solution. This purple solution was filtered through cotton wool and vapor diffused with diethyl ether resulting in deep purple X-ray quality crystals of **8**. The crystals were isolated by filtration and washed with Et_2_O and dried *in vacuo* (0.125 g, 90%). MP: >230 °C. ^1^H-NMR (500 MHz, CD_3_CN): δ 8.96 (s, 6H, H_e_), 8.60 (d, *J* = 7.7 Hz, 6H, H_d_), 8.43 (t, *J* = 7.6 Hz, 6H, H_c_), 7.81–7.75 (m, 6H, H_b_), 7.38 (d, *J* = 5.5 Hz, 6H, H_a_), 7.08–6.87 (m, 12H, H_f_), 5.55–5.51 (m, 12H, H_g_), 4.05 (s, 6H, H_h_). ^13^C-NMR (125 MHz, CD_3_CN): δ 175.5, 159.1, 156.8, 150.2, 142.7, 140.7, 132.4, 131.1, 130.1, 122.5, 40.1. ATR-IR: υ (cm^−1^) 3747, 3605, 3035, 2908, 1613, 1583, 1556, 1500, 1471, 1441, 1414, 1358, 1302, 1258, 1238, 1049. Anal. calcd for C_75_H_60_B_4_F_16_Fe_2_N_12_•(3.5H_2_O): C 54.55, H 4.09, N 10.18%; found: C 54.41, H 4.00, N 10.20%. UV/Vis (CH_3_CN) λ_max_/nm (ε/L mol^−1^cm^−1^) = 237 (7.7 × 10^4^), 324 (3.6 × 10^4^), 571 (1.7 × 10^4^).

### 3.5. General Method for the Molecular Docking Studies

Molecular docking studies were carried out using GOLD 5.1. [96]. The X-ray crystal structures of the Fe(II) complexes (**6a**, **6b**, **6c** and **6f**) were used for the docking experiments with some modifications. The cif formatted files were converted to mol2 files using Mercury (CCDC) and edited to remove any counter ions and to add appropriate atom and bond types using SYBYLX 2.0. The Fe(II) cylinders were docked as rigid molecules into either the major groove of B-DNA (pdb3BSE) [97] or the three-way (triplex) DNA junction (pdb2et0) [29]. The docking cavity of B-DNA was identified as the solvent accessible surface centered on the position of water 209 and was adjacent to the sequence 5'(CAATGTTGC) and 5'(GCAACATTG). The cavity of the triplex DNA was identified as the solvent accessible surface (of the DNA) centered around the Fe(II) atom centered within the triplex.

### 3.6. Yeast Agarose Diffusion Assays

A base layer of Complete Synthetic Medium (CSM, 20 mL) was solidified with agarose (0.6%) in a Petri dish. A 5 mL overlay of the same medium containing *S. cerevisiae* cells (strain ADΔ/pABC3; 100 μL, OD_600_ = 0.4 in CSM, pH 4.8–5) at 45 °C was poured and allowed to solidify. Discs (5 mm diameter; BBL, Becton Dickinson Co., Sparks, MD, USA) containing Fe(II) cylinders (5 μL of 20 mM solution in DMSO; 100 nmol/disc) were placed on the agarose (strain ADΔ/pABC3). The plate was then incubated at 30 °C for 48 h. Amphotericin B (80% Sigma) was used as a positive control (7 μL of 1 mM solution in DMSO; 7 nmol/disc). DMSO was used as a negative control.

### 3.7. General X-ray Experimental Section

X-Ray data were recorded at 89 K using a Bruker APEX II CCD diffractometer using Mo-Kα radiation (*λ* = 0.71073 Å) except for **6f** which was collected at 100 K on crystals mounted on a Hampton Scientific cryoloop at the MX2 beamline of the Australian synchrotron [98]. Absorption corrections were applied by semi-empirical methods (SADABS) [99].The structures were solved by direct methods using SIR97 [100], or SHELXS-97 [101] with the resulting Fourier maps revealing the location of most non-hydrogen atoms. Full-matrix least-squares refinement on *F*^2^ was carried out using SHELXL-97 with most non-hydrogen atoms being refined anisotropically. The non-hydrogen atoms that were isotropically refined are described in the Supporting Information for each structure. The hydrogen atoms were included in calculated positions and were refined as riding atoms with individual (or group, if appropriate) isotropic displacement parameters. The PLATON SQUEEZE [77] procedure was used to treat regions of diffuse solvent and counter anions which could not be sensibly modeled in terms of atomic sites, see Supporting Information. Some of the crystal structures contained disordered components. The ORTEP [102] diagrams have been drawn with 50% probability ellipsoids. Crystal data and collection parameters are given in the Supporting Information. The CIF files CCDC 931616–931621 contain the supplementary crystallographic data for this paper. These data can be obtained free of charge via www.ccdc.ac.uk/conts/retrieving.html (or from the Cambridge Crystallographic Data Centre, 12 Union Road, Cambridge CB2 1EZ, UK; Fax: (+44) 1223-336-033.

*3.8.^1^H-NMR and UV-Vis Histidine Competition Experiments*

^1^H-NMR study of **6a**: Fe(II) cylinder **6a** (0.010 g, 0.006 mmol, 1 eq.), D,L-histidine hydrochloride monohydrate (0.007 g, 0.03 mmol, 6 eq.), and sodium hydrogen carbonate (0.004 g, 0.05 mmol, **8** eq.), were dissolved in a mixture of CD_3_CN and D_2_O (97:3). The resulting suspension was sonicated for few seconds until the histidine and sodium hydrogen carbonate were completely dissolved and then the ^1^H NMR spectrum was recorded. 

^1^H-NMR study of **8**: Fe(II) cylinder **8** (0.010 g, 0.006 mmol, 1 eq.), D,L-histidine hydrochloride monohydrate (0.008 g, 0.04 mmol, 6 eq.), and sodium hydrogen carbonate (0.003 g, 0.04 mmol, 6 eq.), were dissolved in a mixture of CD_3_CN and D_2_O (97:3). The resulting suspension was sonicated for few seconds, until the histidine and sodium hydrogen carbonate were completely dissolved and then ^1^H NMR spectrum was immediately recorded. 

UV-Vis study for **6a** and **8**: The Fe(II) cylinder, either **6a** (0.0050 g, 1 eq.) or **8** (0.0050 g, 1 eq.), was dissolved in 10 mL of acetonitrile:water (97:3) giving a stock solution. D,L-Histidine hydrochloride monohydrate (0.0035 g, 6 eq.) and sodium hydrogen carbonate (0.0025 g, 6 eq.) were also dissolved in 10 mL of acetonitrile/water (97:3). The stock solution containing Fe(II) cylinders (5 mL), either **6a** or **8**, was added to the stock solution containing histidine and sodium hydrogen carbonate (5 mL), mixed thoroughly and UV-Vis spectra were recorded immediately.

## 4. Conclusions

A series of metallosupramolecular [Fe_2_L_3_]^4+^ “click” cylinders have been synthesized in excellent yields (90%–95%) from [Fe(H_2_O)_6_](BF_4_)_2_ and bis(bidentate) pyridyl-1,2,3-triazole ligands. All complexes were characterized by elemental analysis, IR, UV-vis, ^1^H-, ^13^C- and DOSY-NMR spectroscopies. In four cases the structures were confirmed by X-ray crystallography. Molecular modeling studies indicated that some of these complexes were of suitable size and shape to bind both duplex and triplex DNA in a similar fashion to some related biologically active iron(II) helicates. In contrast to the previously reported pyridylimine based iron(II) helicates, the “click” cylinders display no antifungal activity against the yeast *S. cerevisiae. *This is probably due to the poor stability of the “click” complexes in the presence of DMSO and the amino acid histidine found in the biological test media. The Fe(II) “click” cylinders rapidly decomposed in these media. The high lability of these Fe(II) “click” complexes is due to two factors; 1) the weaker σ-donor ability of the ligand’s 1,2,3-triazole units, and 2) lone pair-lone pair repulsion between the uncoordinated N2 nitrogen atoms of the “click” ligands when they are in the *fac*-arrangement found in the cylinder structures. Exchanging the labile Fe(II) for more kinetically inert metal ions may enable the development of more robust biologically active metallosupramolecular “click” cylinders. Efforts towards these types of architectures are underway.

## Supplementary Materials

Supplementary materials can be accessed at: http://www.mdpi.com/1420-3049/18/6/6383/s1.
