# Supplementary material for: [Fe2L3]4+ Cylinders Derived from Bis(bidentate) 2-Pyridyl-1,2,3-triazole “Click” Ligands: Synthesis, Structures and Exploration of Biological Activity
Source: Molecules. 2013 May 29;18(6):6383–407. doi: 10.3390/molecules18066383 (PMC6290563; doi:10.3390/molecules18066383)

Supplementary Materials

1. NMR Assignment Schemes

Scheme 1. ¹H-NMR assignments for model Fe(II) complexes **5a** and **5b**.

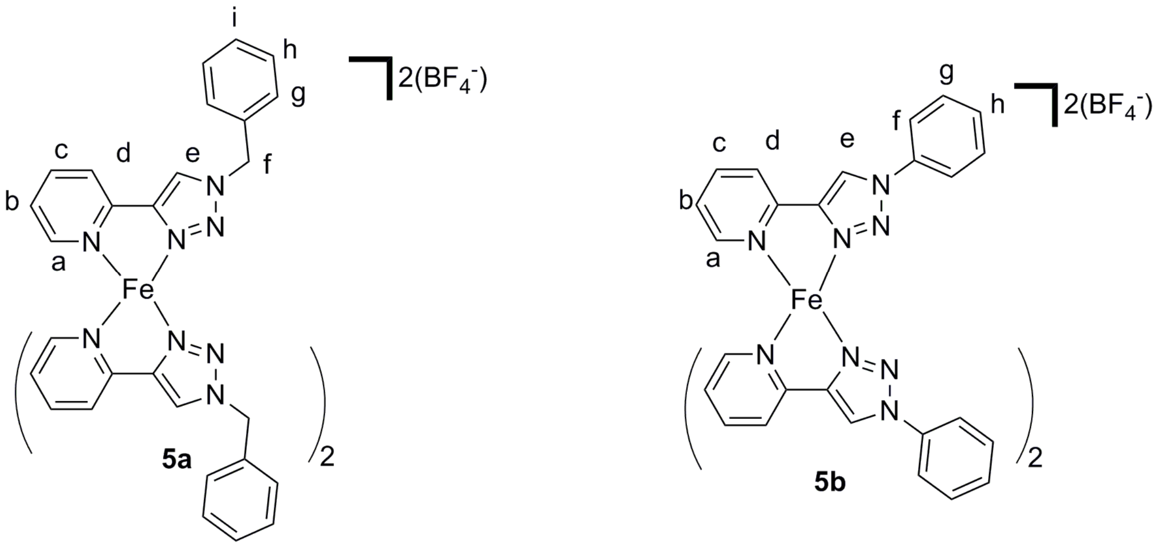

Scheme 2. ^{1}H-NMR assignments for Fe(II) cylinders **6a**–**f** and **8**.

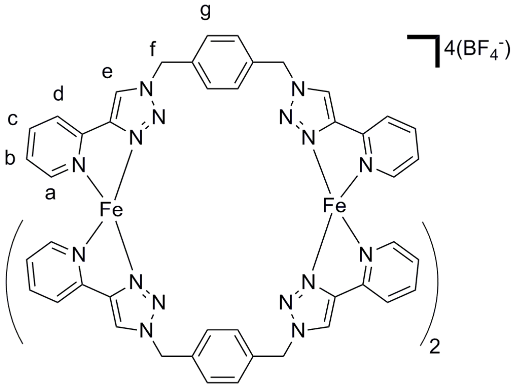

6a

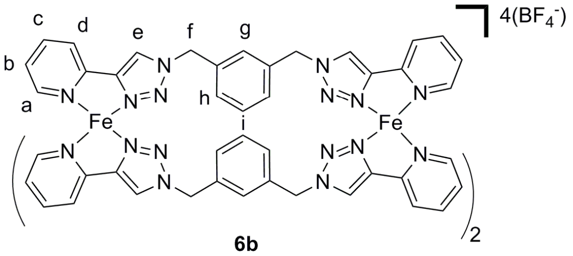

6b

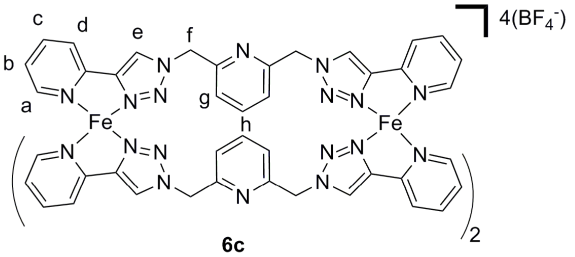

6c

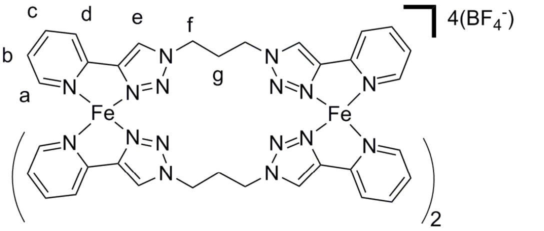

6d

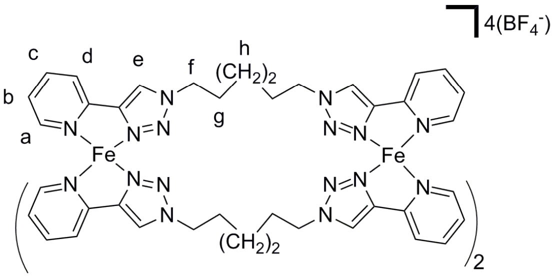

6e

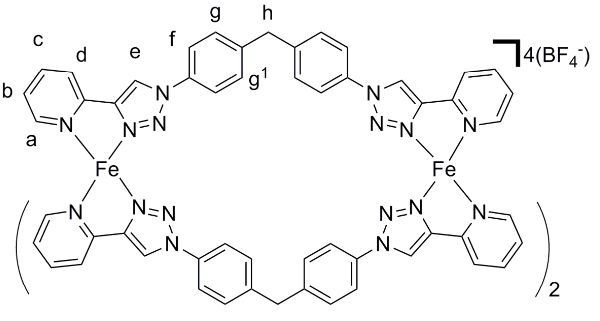

6f

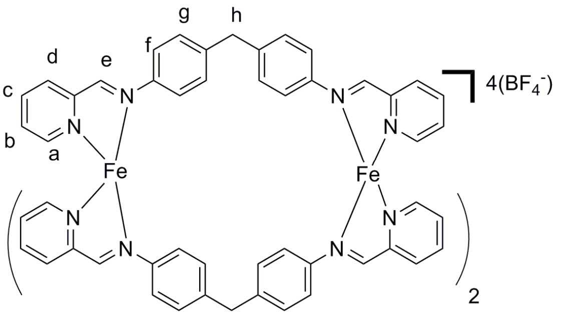

8

2. NMR SpectraFigure S1. Partial ^{1}H-NMR (500 MHz, CD_3CN, 298 K) of **5a**.

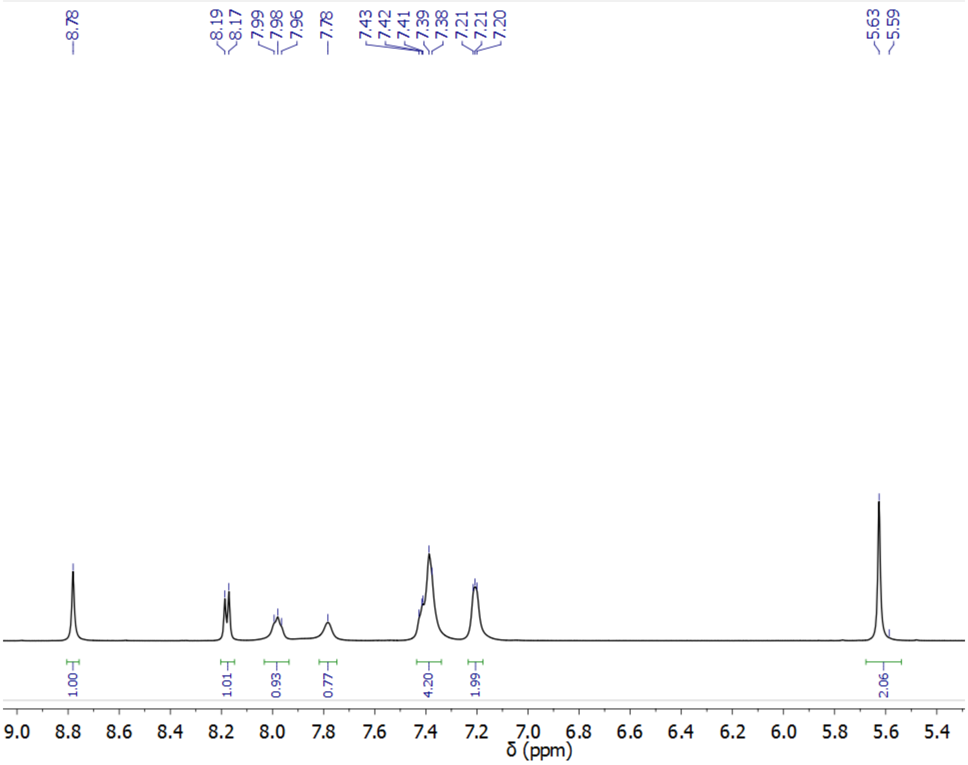

Figure S2. Partial ^{1}H-NMR (500 MHz, CD_3CN, 298 K) of a) the ligand **4a** and b) the Fe(II) model complex **5a**.

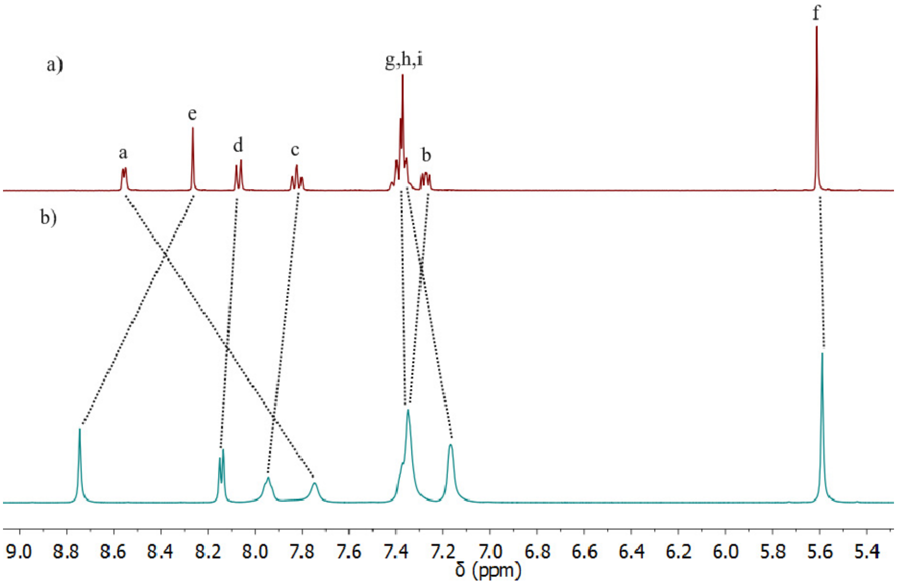

Figure S3. Partial ^{13}C-NMR (125 MHz, CD_3CN, 298 K) of the Fe(II) model complex **5a**.

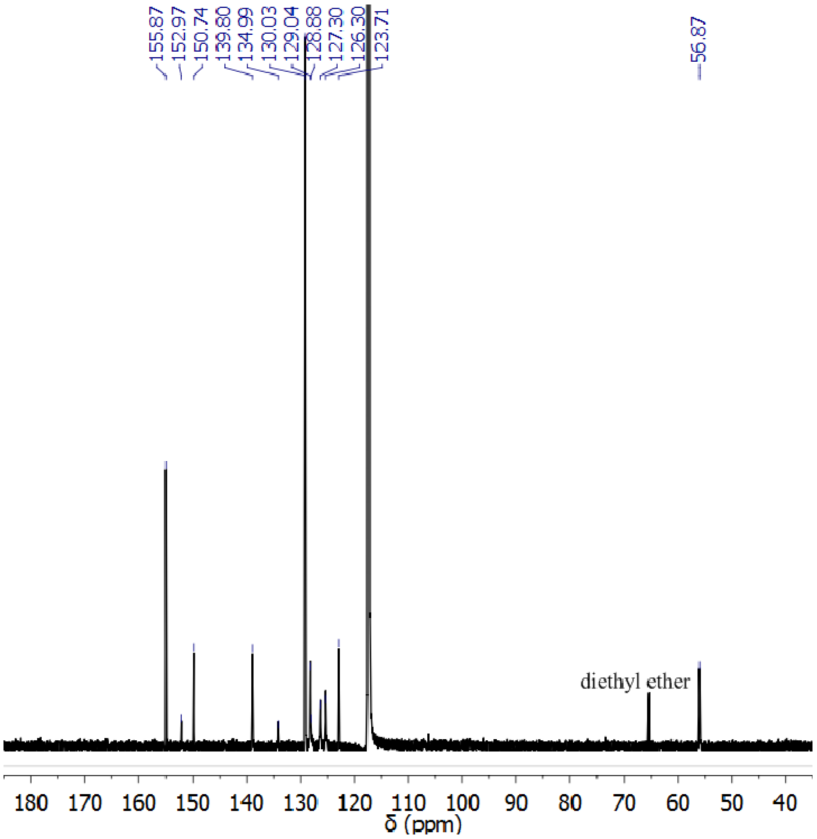

Figure S4. Partial ^{1}H-NMR (500 MHz, CD_3CN, 298 K) of **5b**.

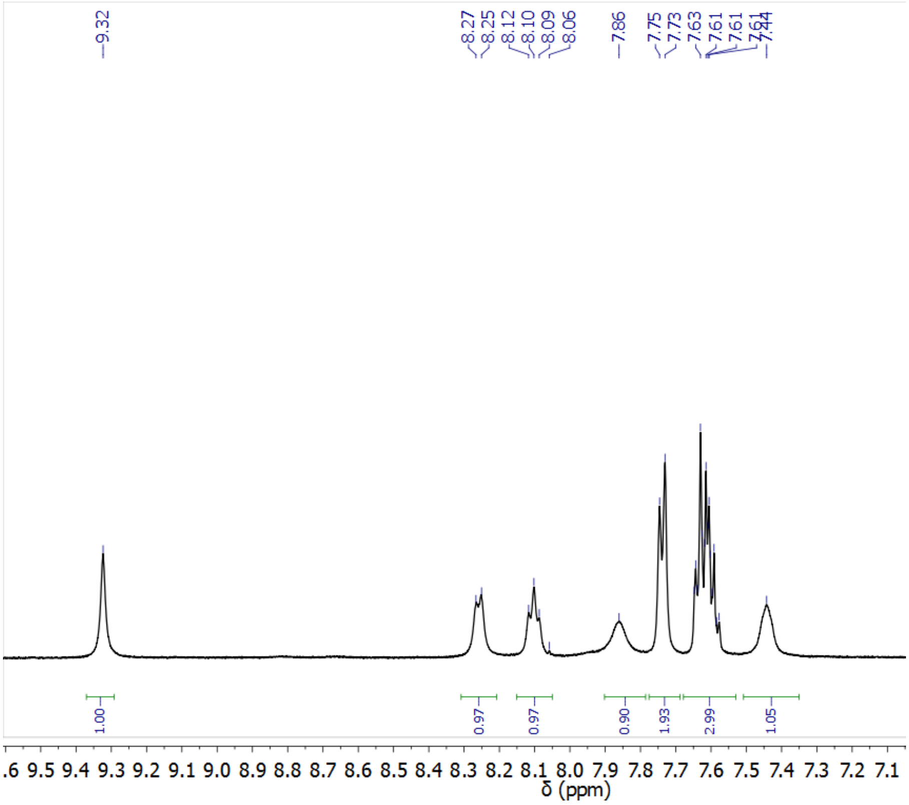

Figure S5. Partial ^{1}H-NMR (500 MHz, CD_3CN, 298 K) of a) the ligand **4b** and b) the Fe(II) model complex **5b**.

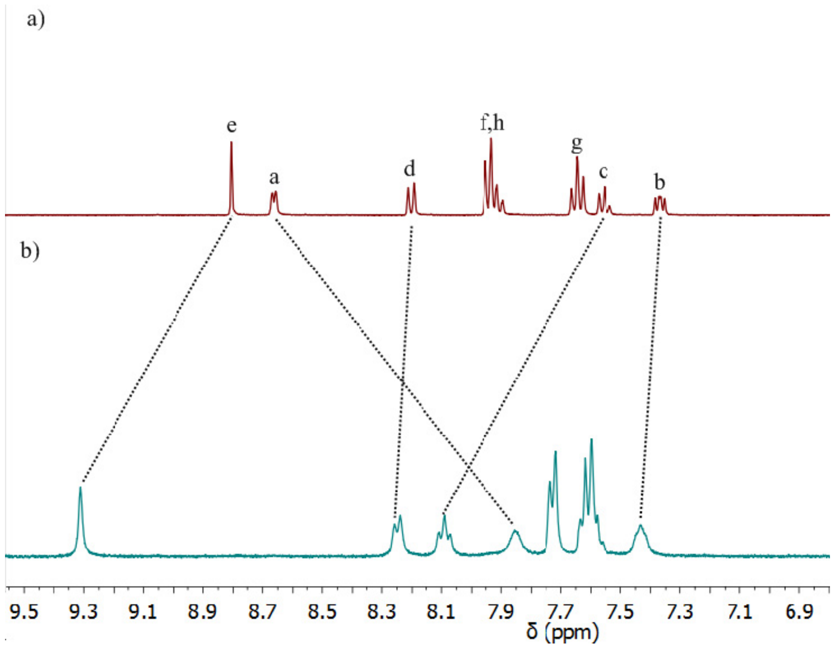

Figure S6. Partial ^{13}C-NMR (125 MHz, CD_3CN, 298 K) of the Fe(II) model complex **5b**.

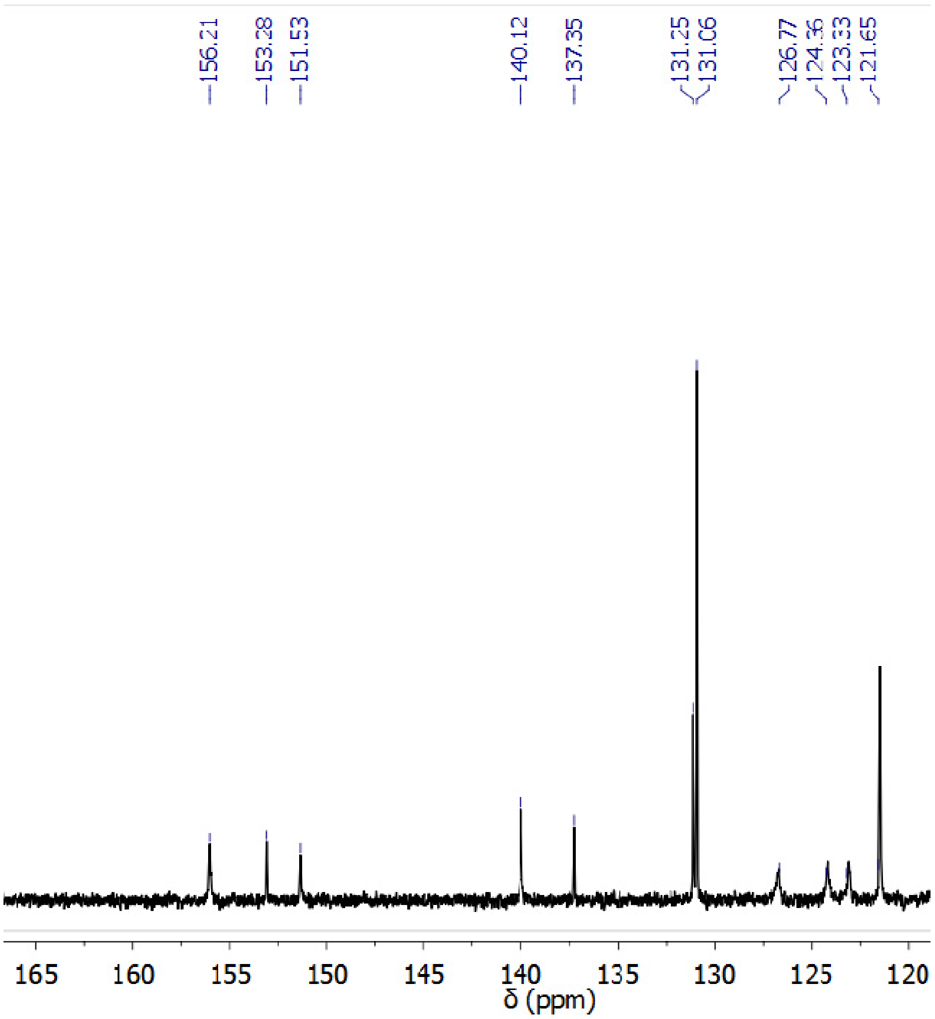

Figure S7. Partial ^{1}H-NMR (500 MHz, CD_3CN, 298 K) of **6a**.

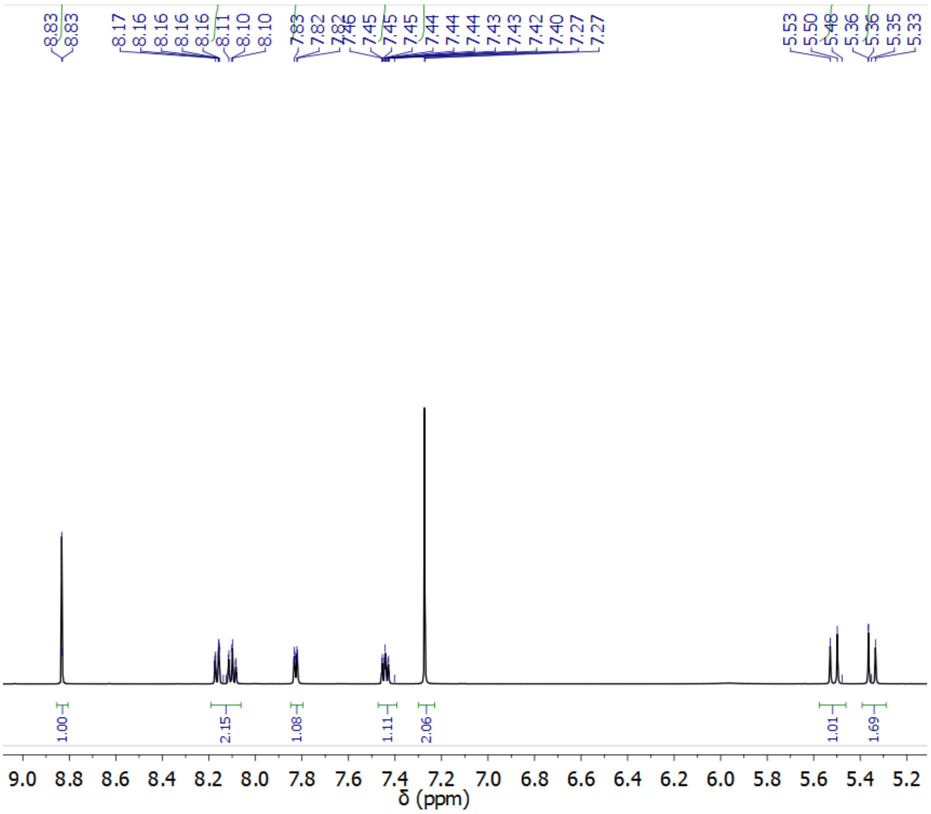

Figure S8. Partial ^{1}H-NMR (500 MHz, CD_3CN, 298 K) of a) the ligand **3a** and b) the Fe(II) cylinder **6a**.

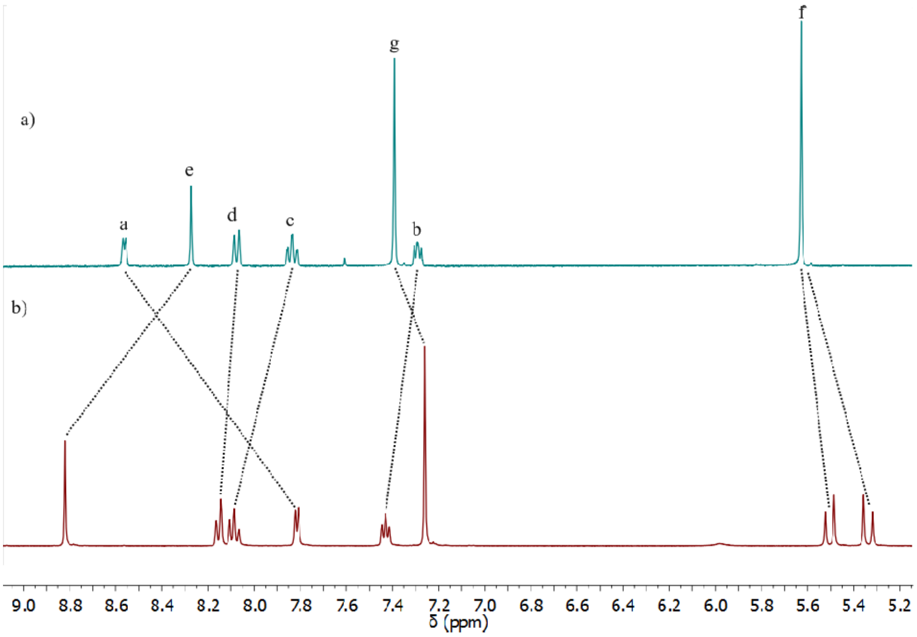

Figure S9. Partial ^{13}C-NMR (125 MHz, CD_3CN, 298 K) of **6a**.

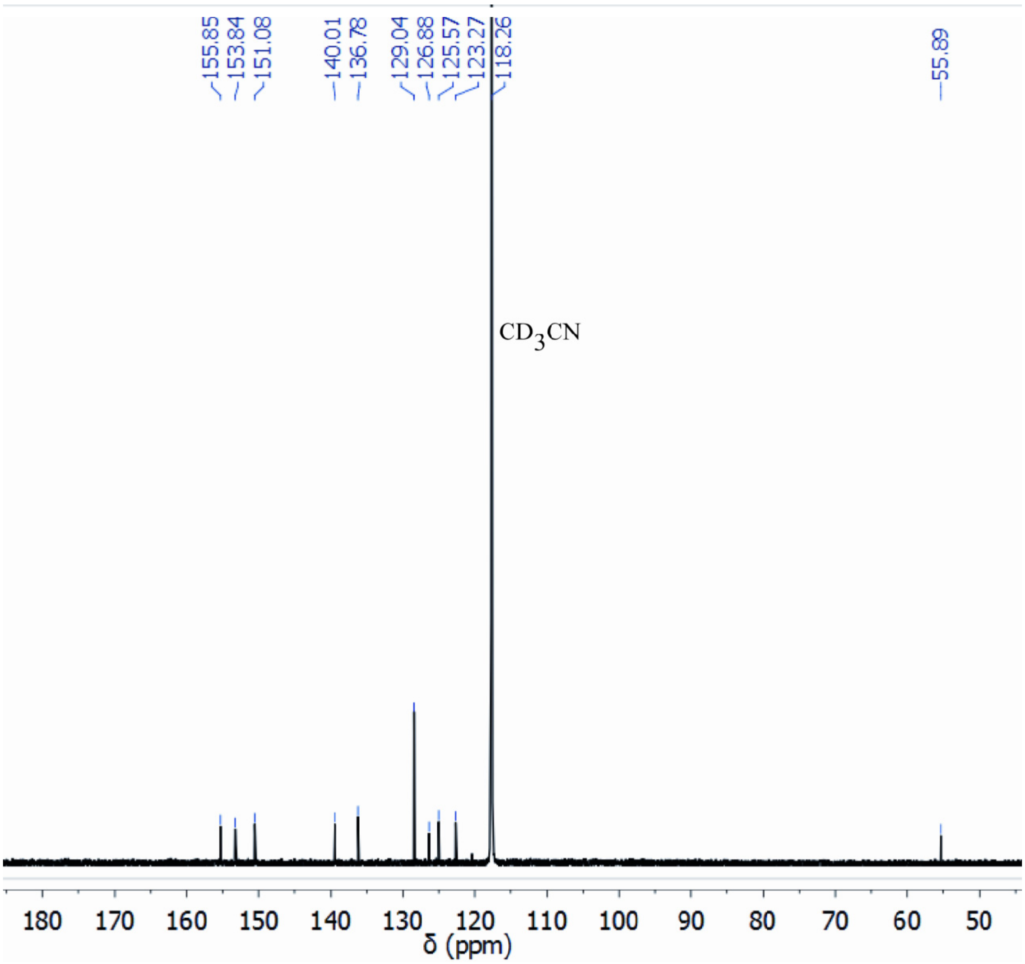

Figure S10. Partial ^{1}H-NMR (500 MHz, CD_3CN, 298 K) of **6b**.

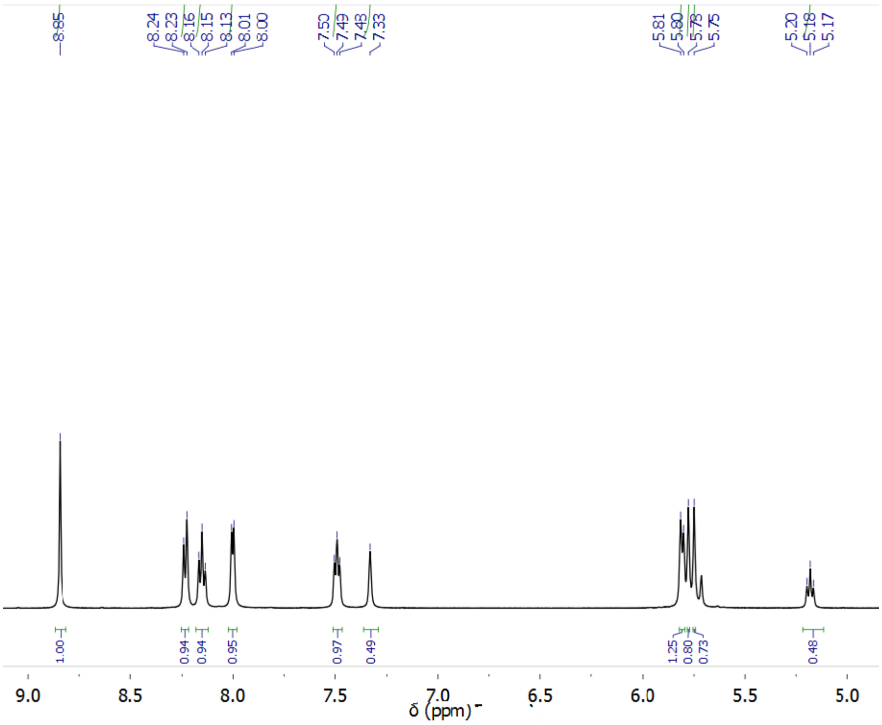

Figure S11. Partial ^{1}H-NMR (500 MHz, CD_3CN, 298 K) of a) the ligand **3b** and b) the Fe(II) cylinder **6b**.

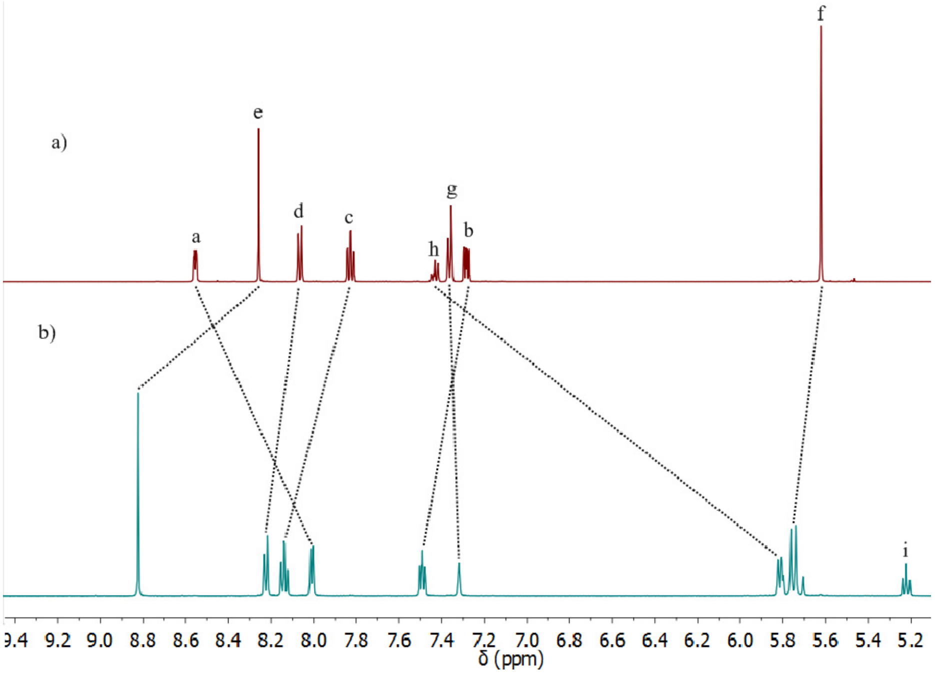

Figure S12. Partial ^{13}C-NMR (125 MHz, CD_3CN, 298 K) of **6b**.

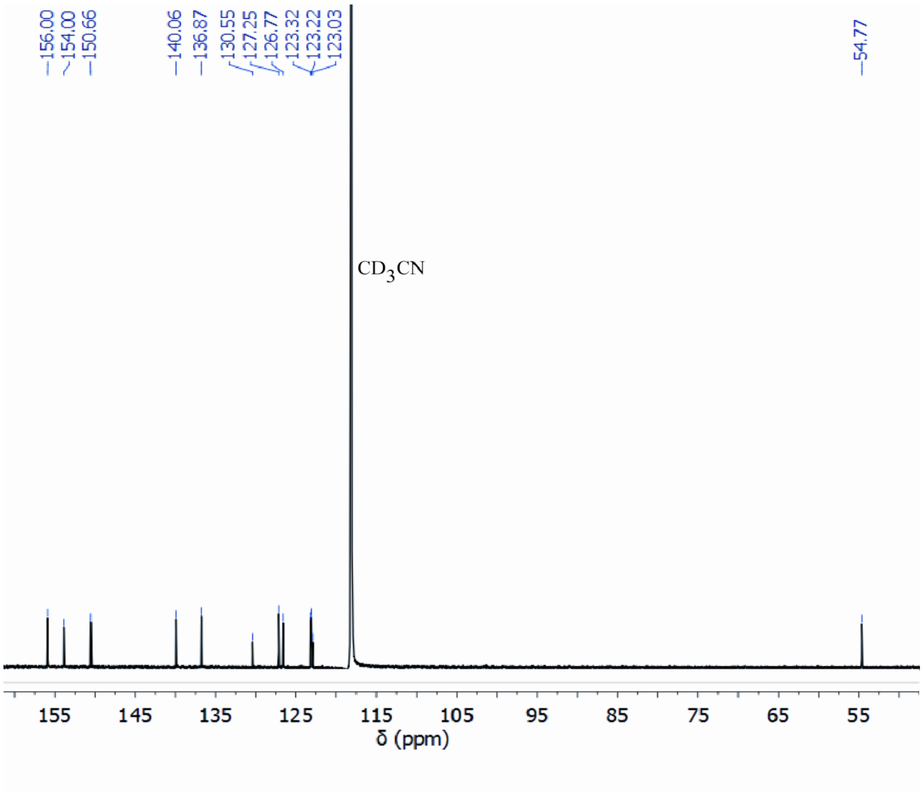

Figure S13. Partial ^{1}H-NMR (500 MHz, CD_3CN, 298 K) of **6c**.

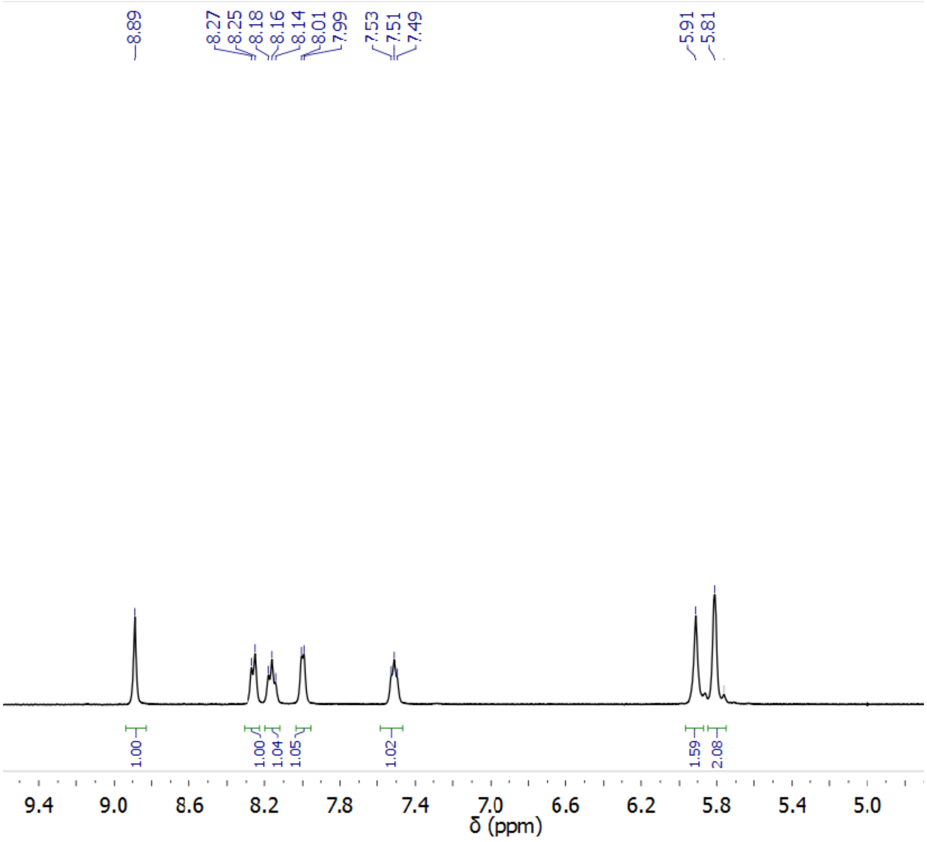

Figure S14. Partial ^{1}H-NMR (500 MHz, CD_3CN, 298 K) of a) the ligand **3c** and b) the Fe(II) cylinder **6c**.

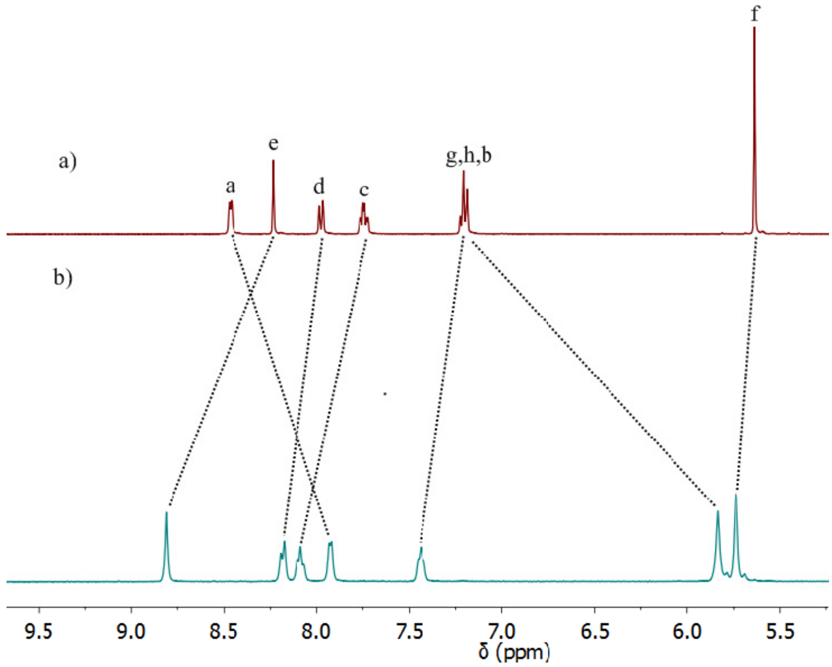

Figure S15. Partial ^{13}C-NMR (125 MHz, CD_3CN, 298 K) of **6c**.

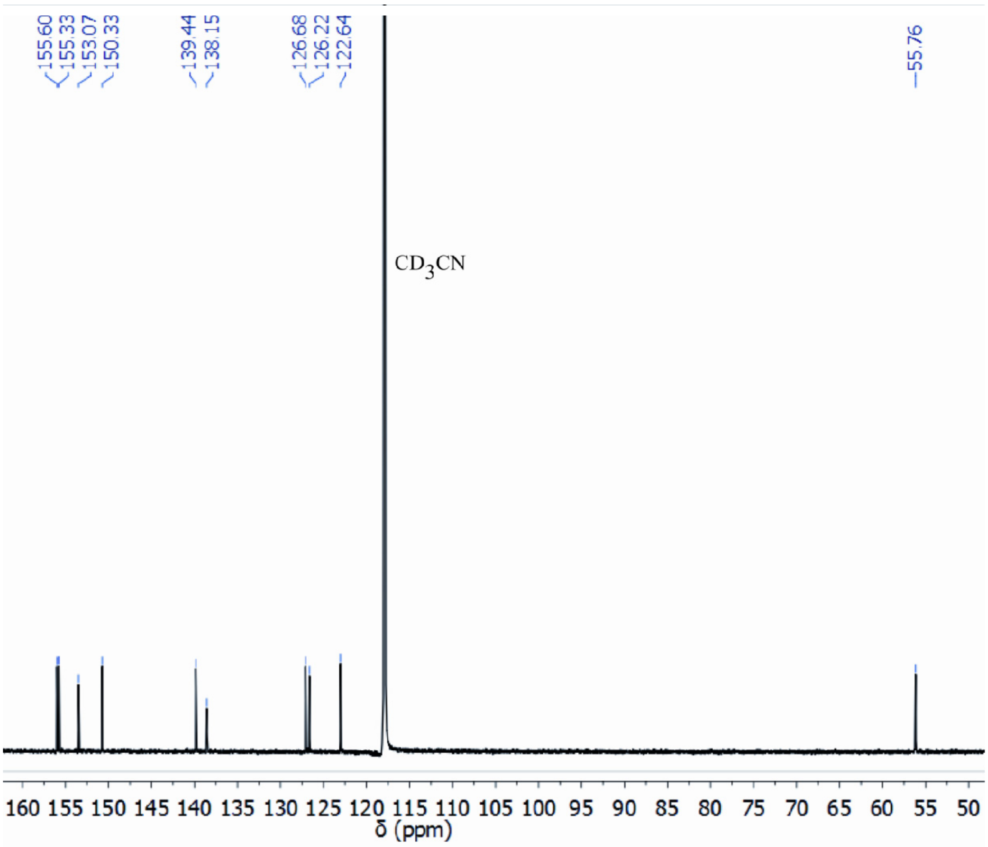

Figure S16. Partial ^{1}H-NMR (500 MHz, CD_3CN, 298 K) of **6d**.

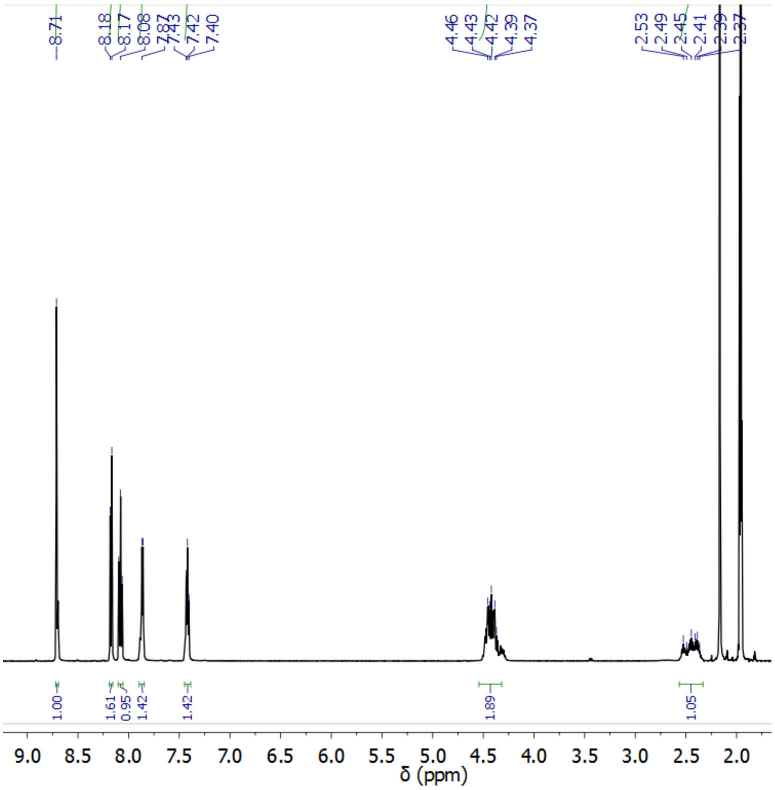

Figure S17. Partial ^{1}H-NMR (500 MHz, CD_3CN, 298 K) of a) the ligand **3d** and b) the Fe(II) cylinder **6d**.

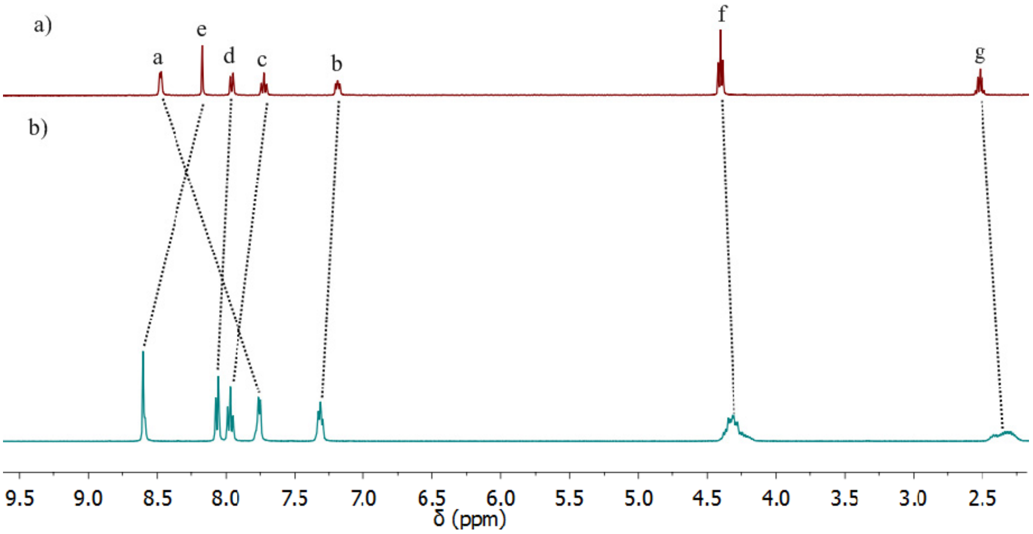

Figure S18. Partial ^{1}H-NMR (500 MHz, CD_3CN, 298 K) of **6e**.

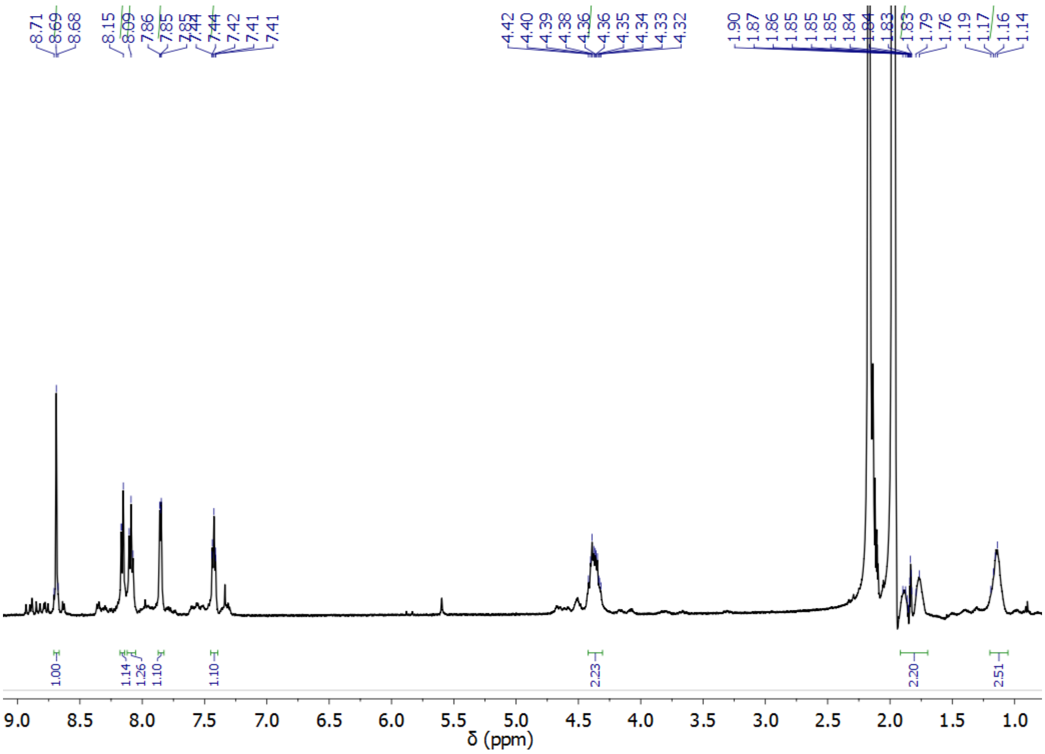

Figure S19. Partial ^{1}H-NMR (500 MHz, CD_3CN, 298 K) of a) the ligand **3e** and b) the Fe(II) cylinder **6e**.

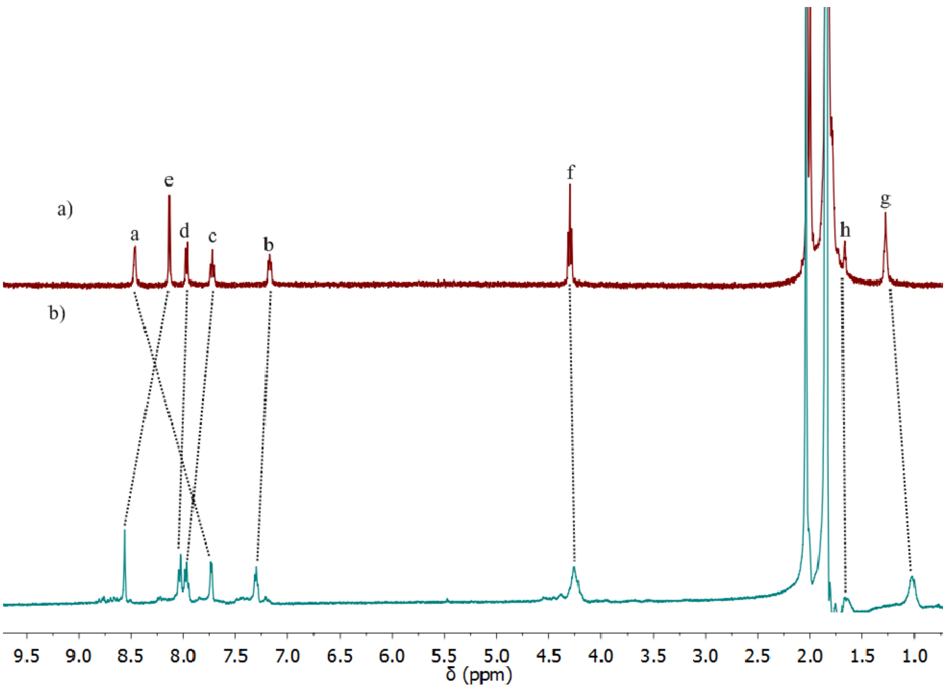

Figure S20. Partial ^{1}H-NMR (500 MHz, CD_3CN, 298 K) of **6f**.

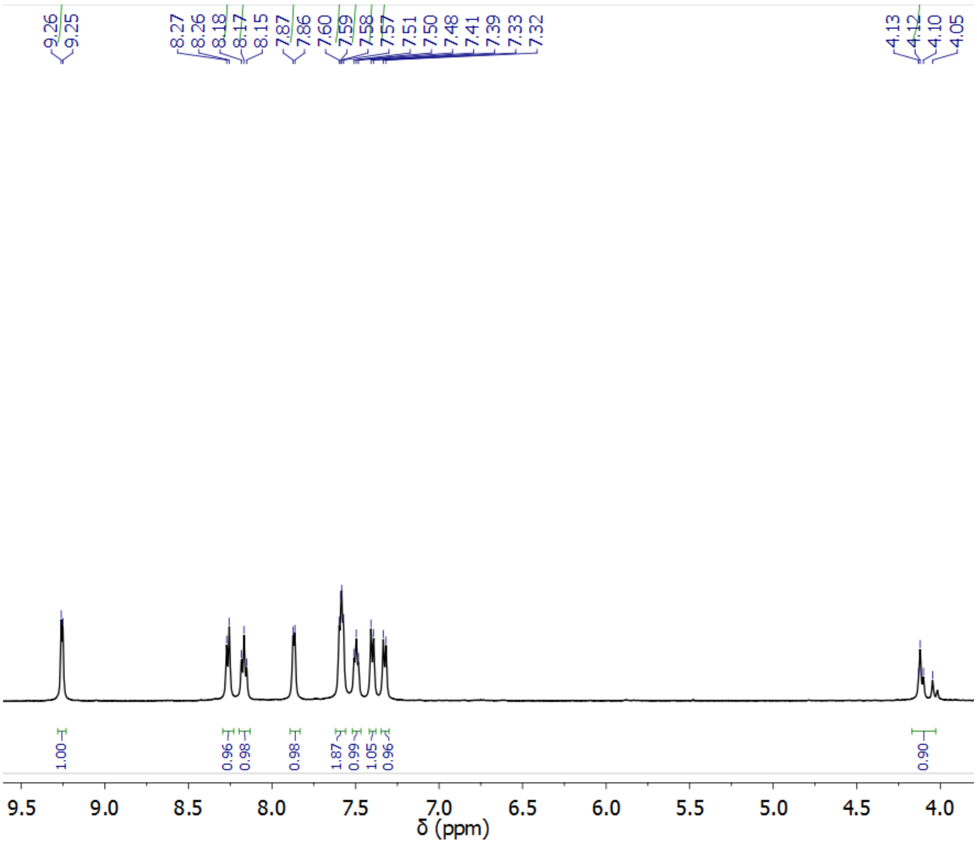

Figure S21. Partial ^{1}H-NMR (500 MHz, CD_3CN, 298 K) of a) the ligand **3f** and b) the mixture *meso*- and *rac*-Fe(II) cylinder **6f**.

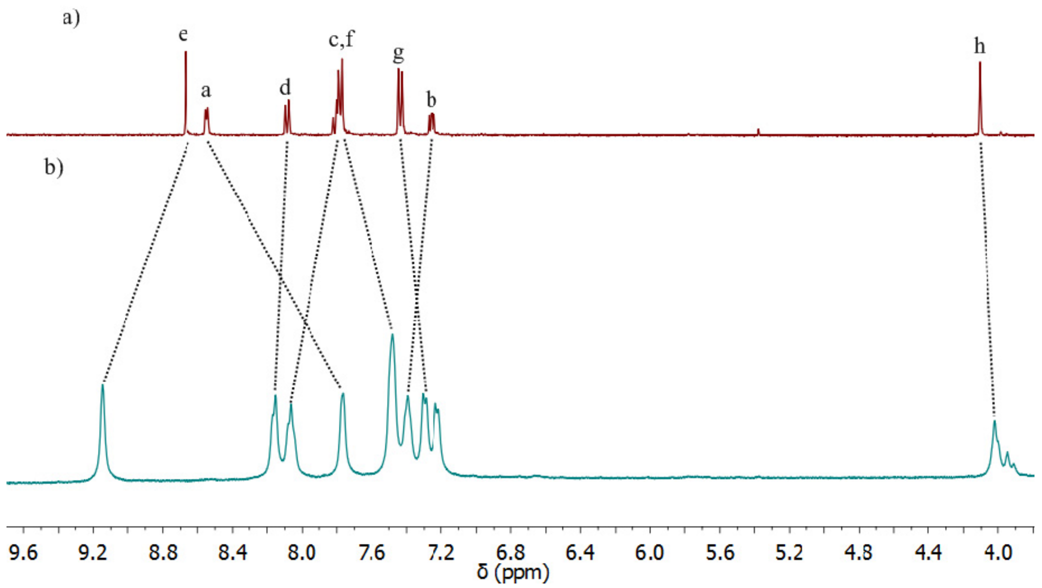

Figure S22. Partial ^{13}C-NMR (125 MHz, CD_3CN, 298 K) of **6f**.

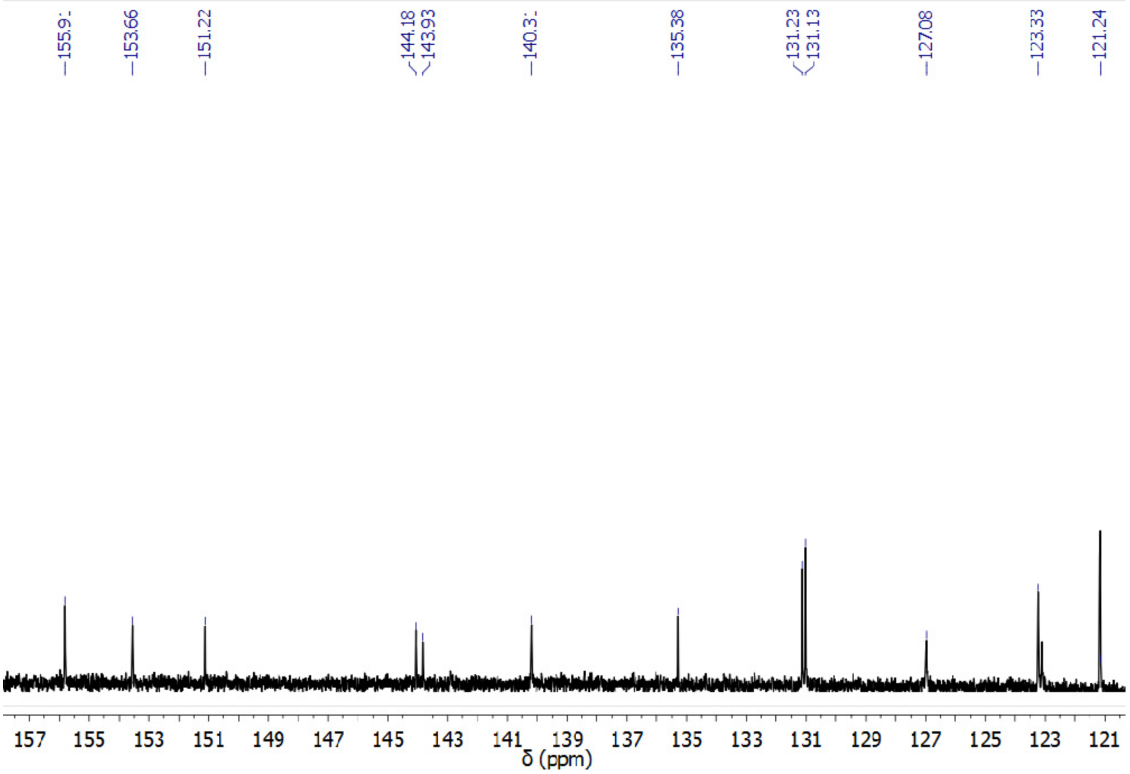

Figure S23. Partial ^{1}H-NMR (500 MHz, CD_3CN, 298 K) of **8**.

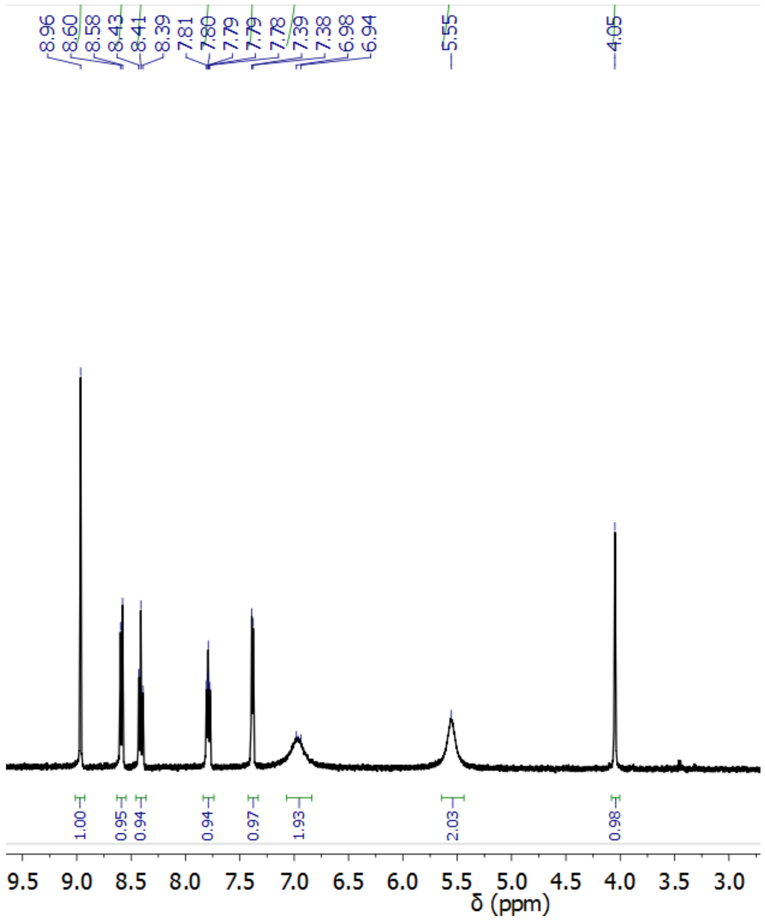

Figure S24. Partial ^{1}H-NMR (500 MHz, CD_3CN, 298 K) of a) the ligand **7** and b) the Fe(II) cylinder **8**.

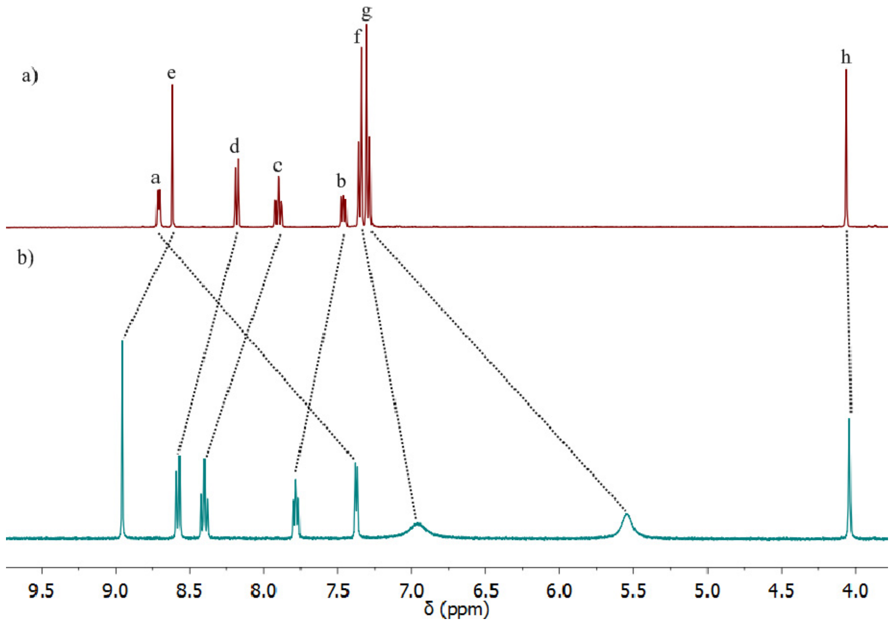

Figure S25. Partial ^{13}C-NMR (125 MHz, CD_3CN, 298 K) of **8**.

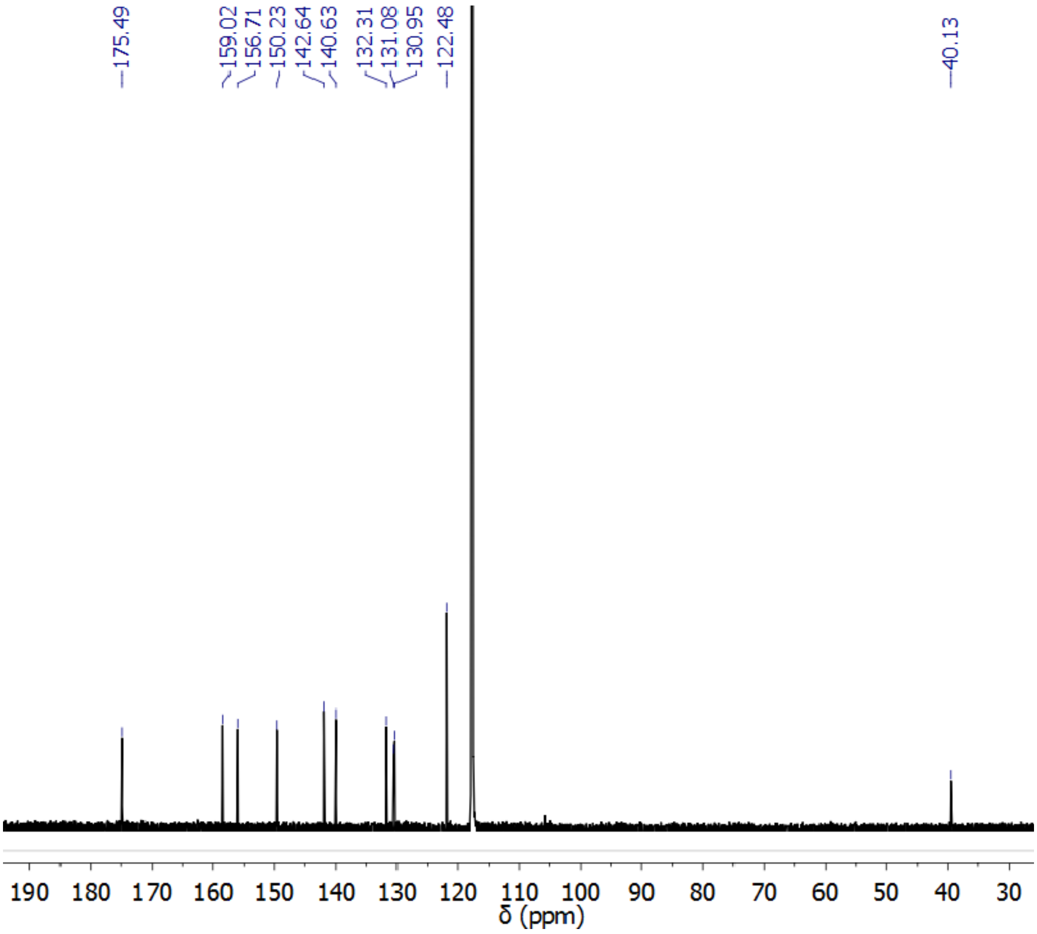

3. ^{1}H DOSY NMR Spectra

Figure S26. DOSY NMR spectra (500 MHz, CD_3CN, 298 K) recorded for the ligand **3a** (top) and the Fe(II) cylinder **6a** (bottom).

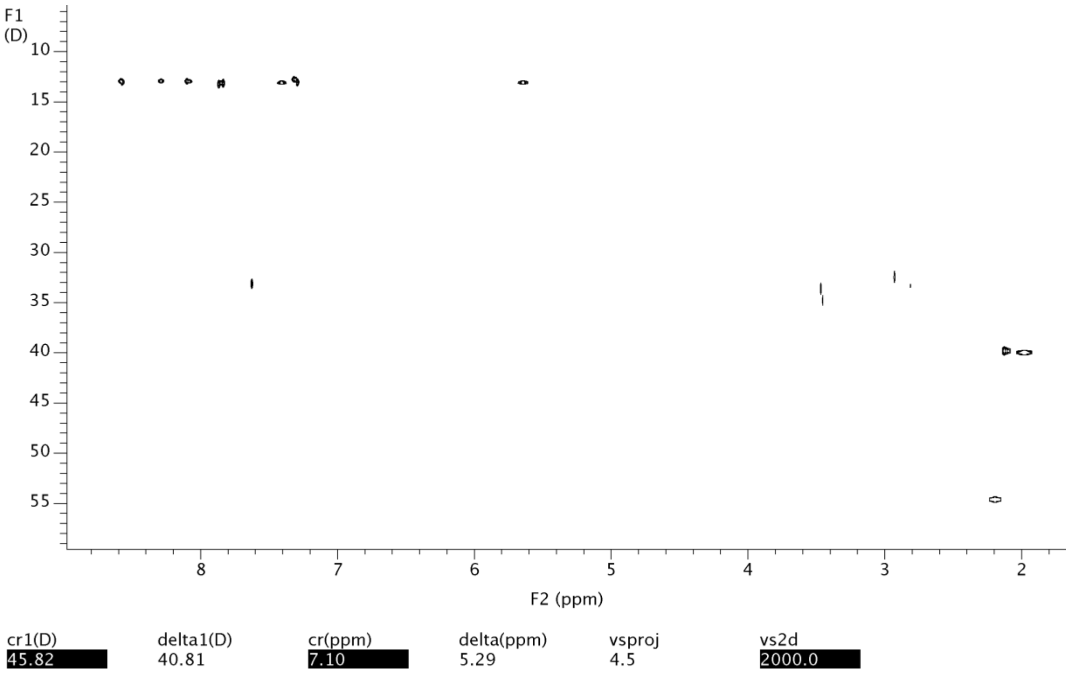

Figure S26. *Cont.*

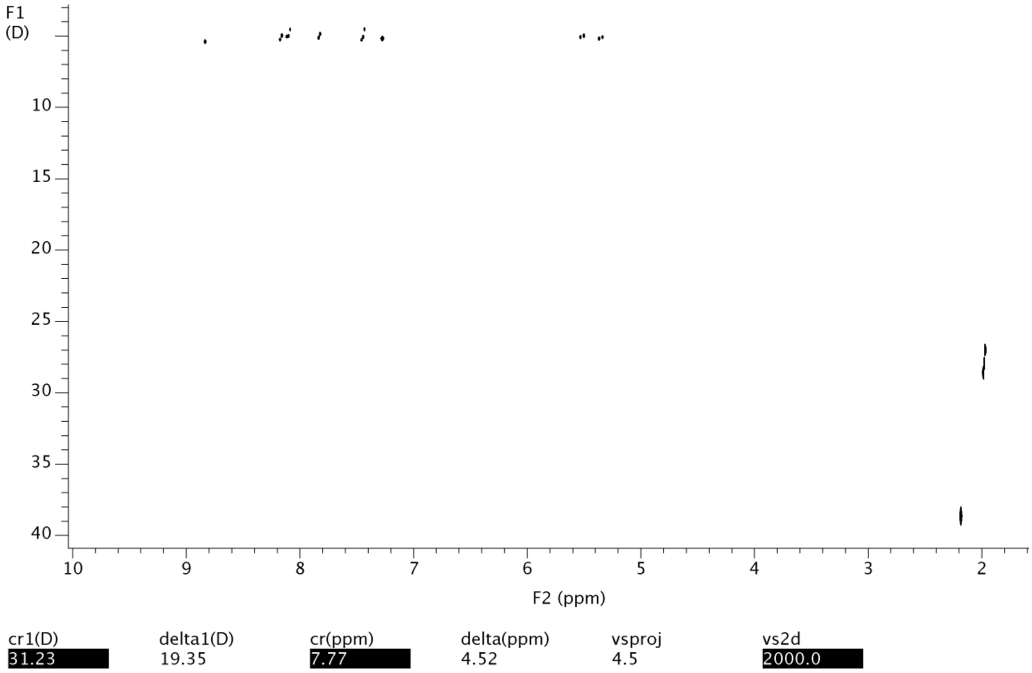

Figure S27. DOSY NMR spectra (500 MHz, CD_3CN, 298 K) recorded for the ligand **3d** (top) and the Fe(II) cylinder **6d** (bottom).

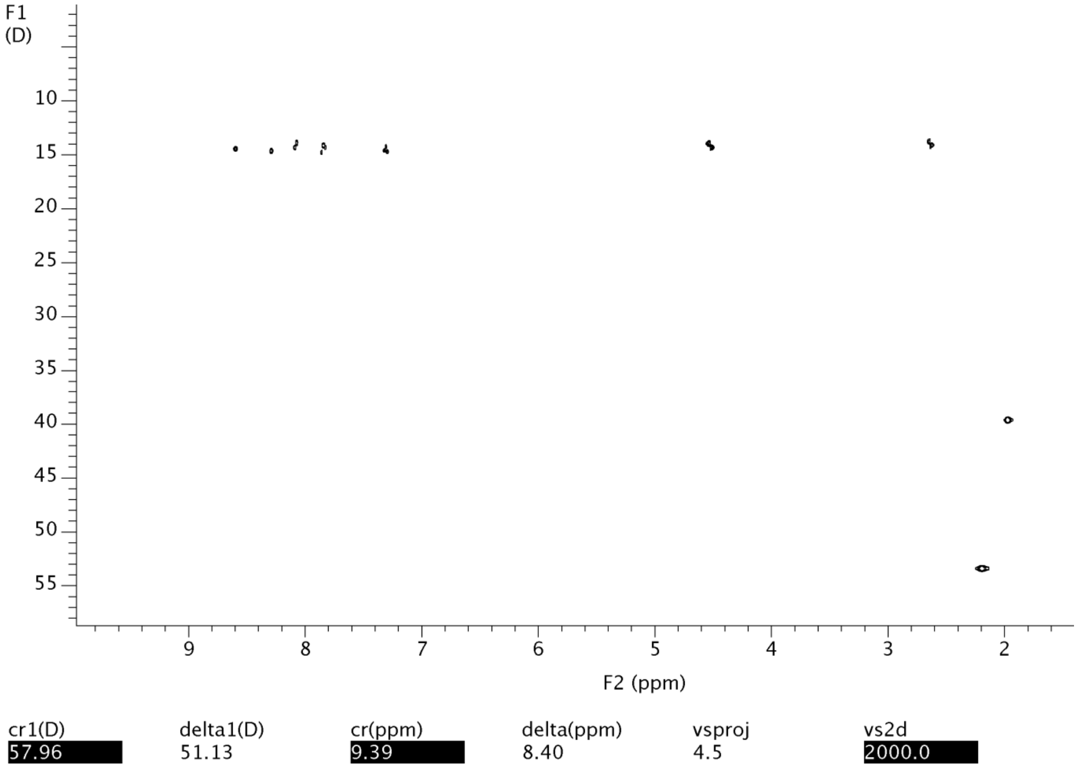

Figure S27. *Cont.*

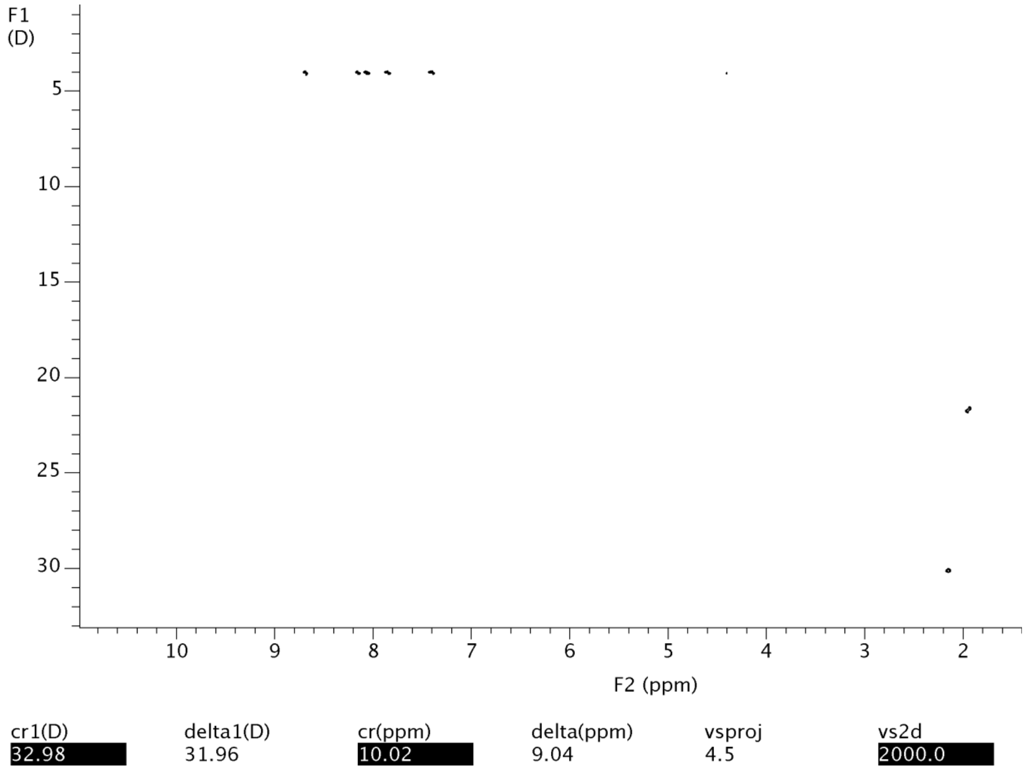

Figure S28. DOSY NMR spectra (500 MHz, CD_3CN, 298 K) recorded for the ligand **3e** (top) and the Fe(II) cylinder **6e** (bottom).

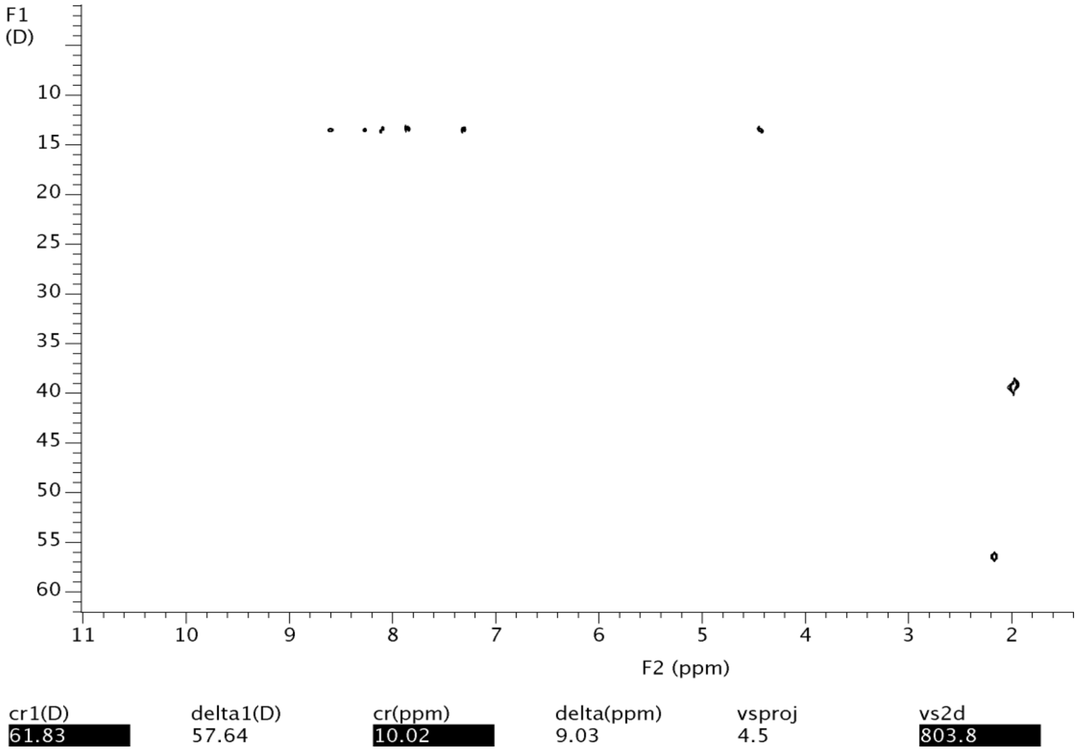

Figure S28. *Cont.*

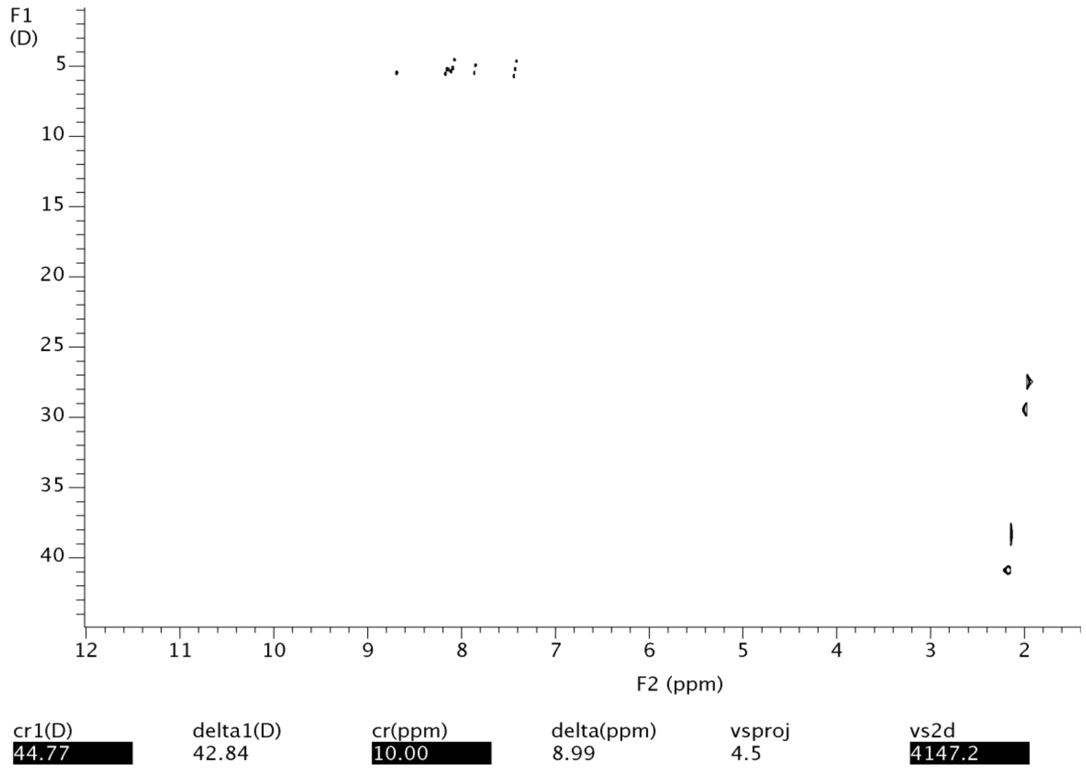

Figure S29. DOSY NMR spectra (500 MHz, CD_3CN, 298 K) recorded for the ligand **3f** (top) and the Fe(II) cylinder **6f** (bottom).

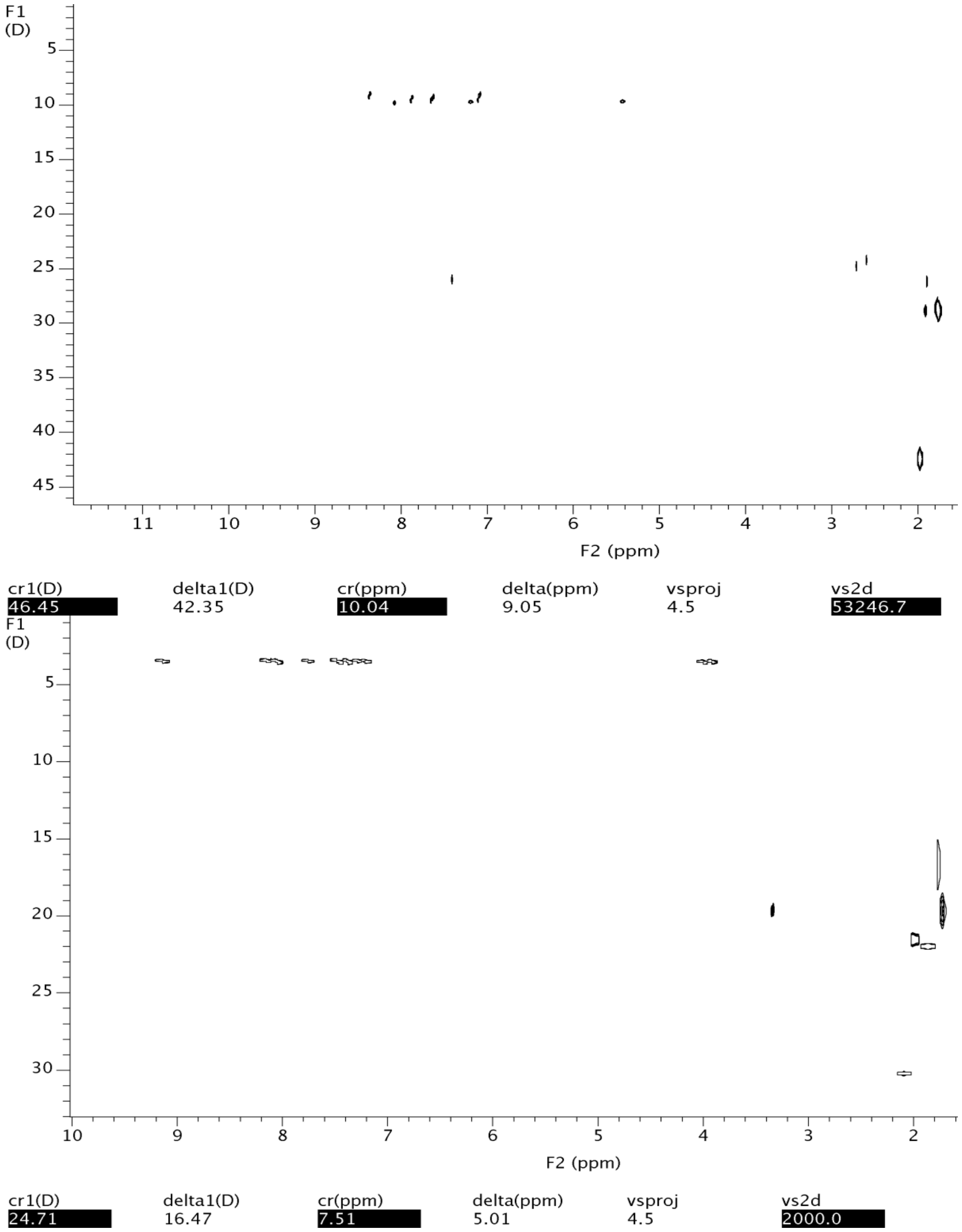

Figure S30. DOSY NMR spectra (500 MHz, CD_3CN, 298 K) recorded for the ligand **7** (top) and the Fe(II) cylinder **8** (bottom).

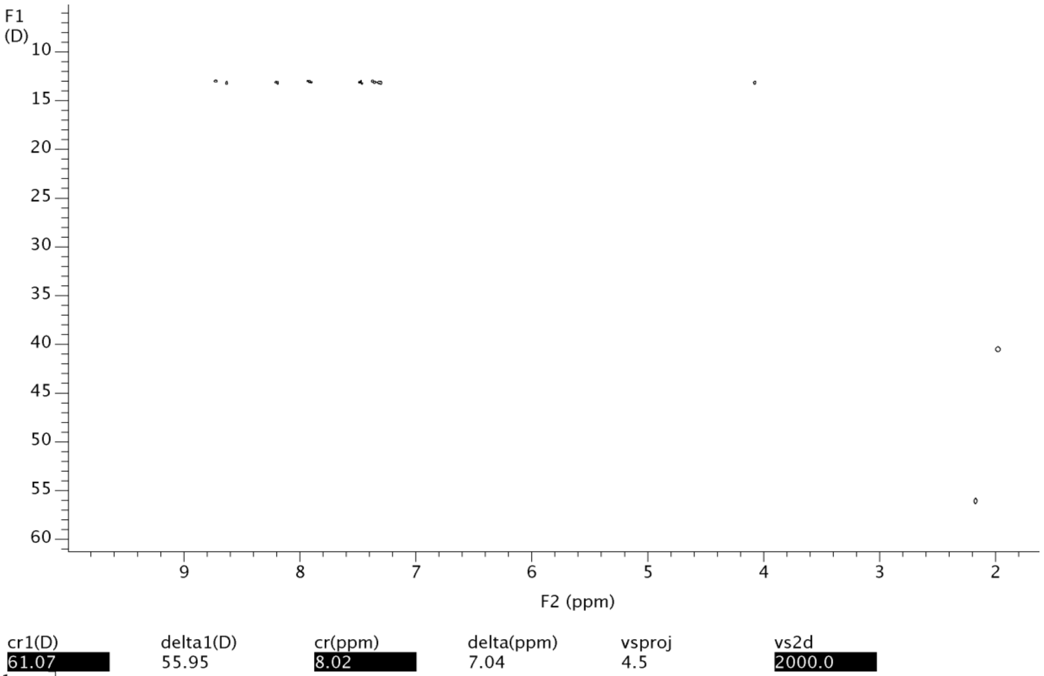

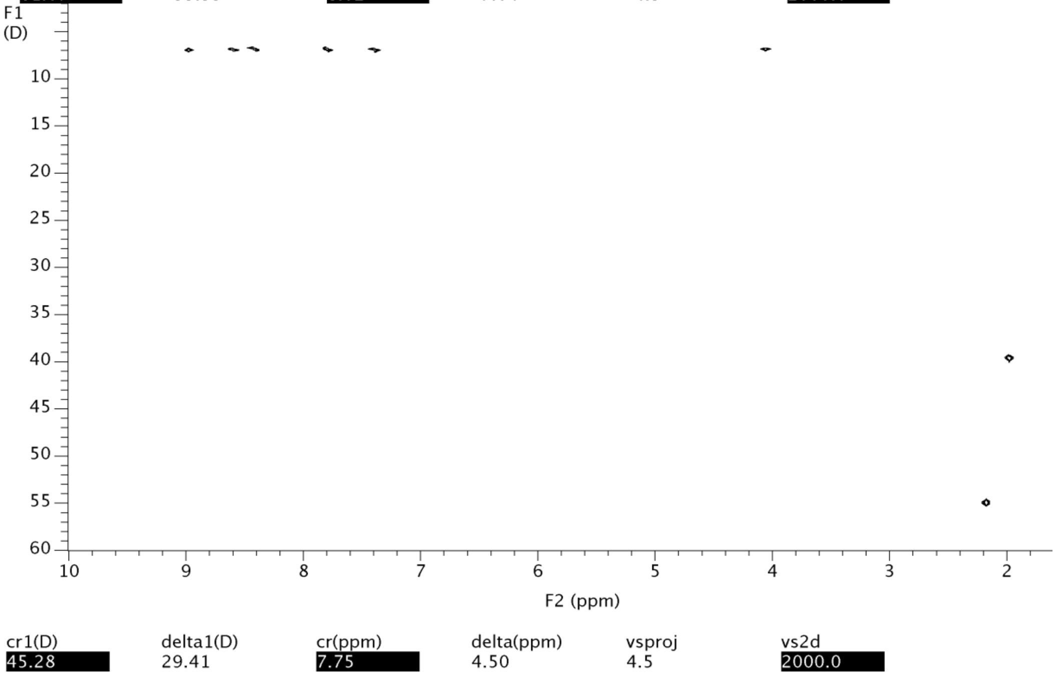

4. X-ray Structure ViewsFigure S31. (**a**) A labelled ORTEP diagram and (**b**) space filling representation of the cation of **6b**. The thermal ellipsoids are shown at the 50% probability level. Selected bond lengths (Å) and angles (°) for **6b**; Fe1-Fe1 12.182, Fe-N1 1.998 (3), Fe-N2 1.923(3); N1-Fe-N2 81.19 (13).

(a)

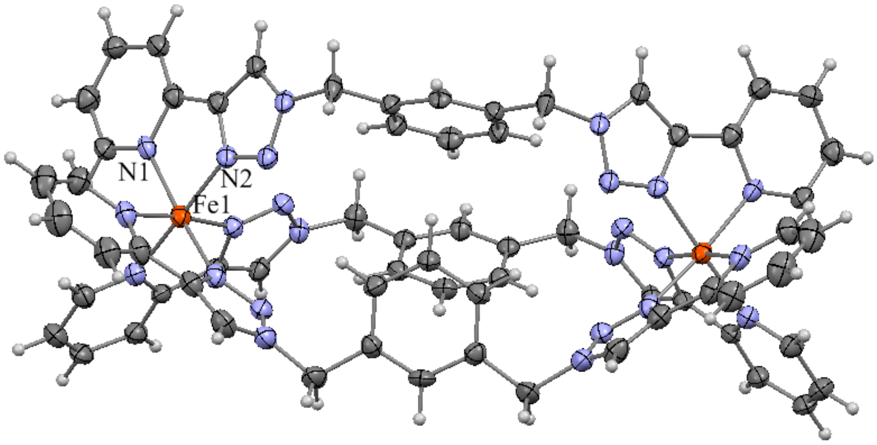

(b)

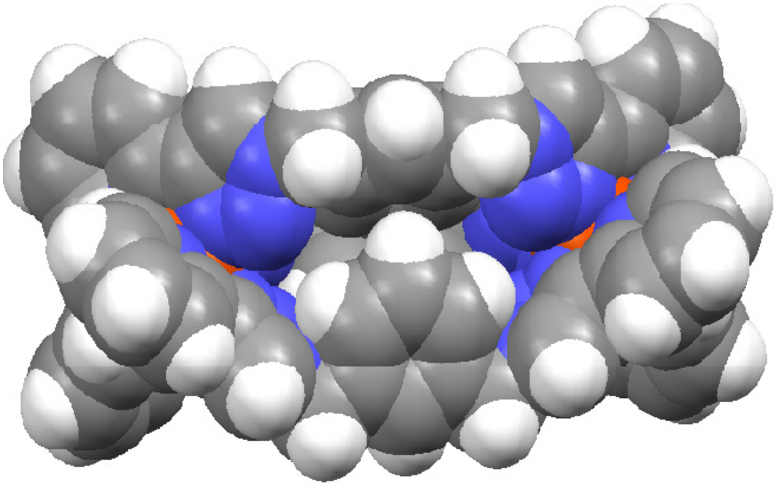

Figure S32. (**a**) A labelled ORTEP diagram and (**b**) space filling representation of the cation of **6c**. The thermal ellipsoids are shown at the 50% probability level. Selected bond lengths (Å) and angles (°) for **6c**; Fe1-Fe2 12.234, Fe1-N1 2.001 (4), Fe1-N2 1.944(3); N1-Fe-N2 81.05(15).

a)

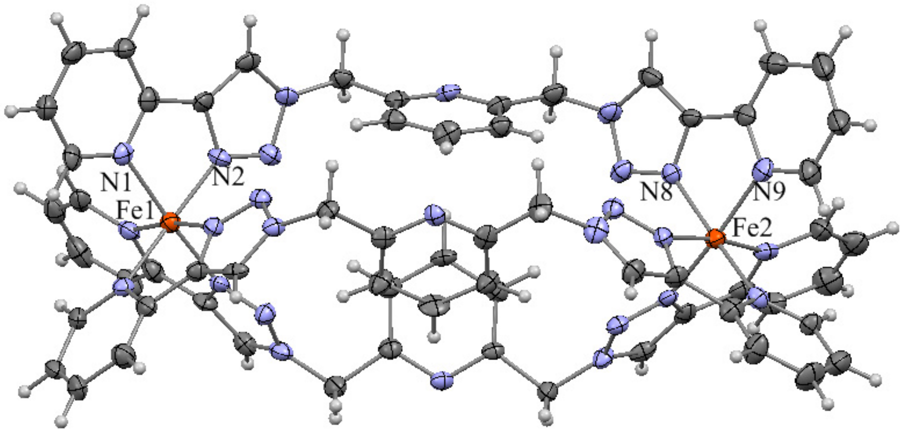

b)

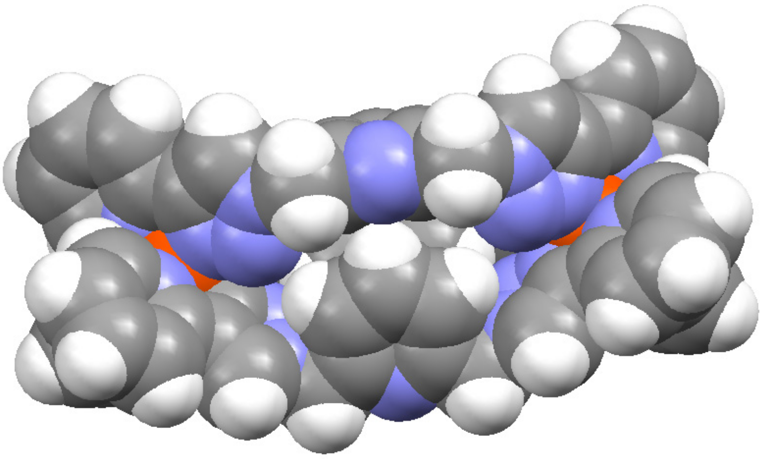

Figure S33. (**a**) A labelled ORTEP diagram and (**b**) space filling representation of the cation of **6f**. The thermal ellipsoids are shown at the 50% probability level. Selected bond lengths (Å) and angles (°) for **6f**; Fe1-Fe 2 14.579, Fe1-N1 1.938, Fe1-N6 2.009; N1-Fe1-N6 81.30.

a)

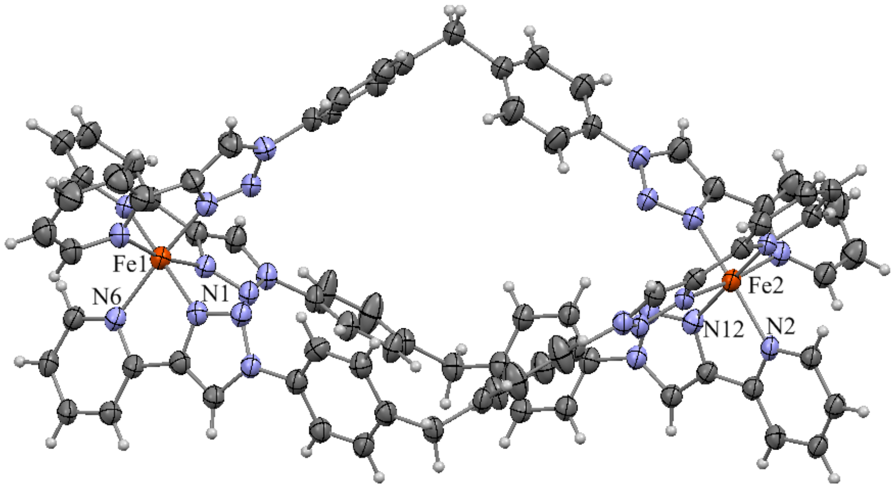

b)

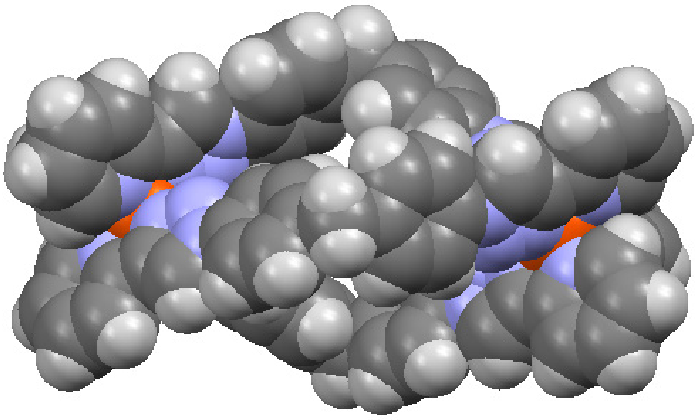

Figure S34. (**a**) A labelled ORTEP diagram and (**b**) space filling representation of the cation of **8**. The thermal ellipsoids are shown at the 50% probability level. Selected bond lengths (Å) and angles (°) for **8**; Fe1-Fe 2 11.246, Fe1-N1 1.994(4), Fe1-N3 2.010(4); N1-Fe-N3 80.65(15).

a)

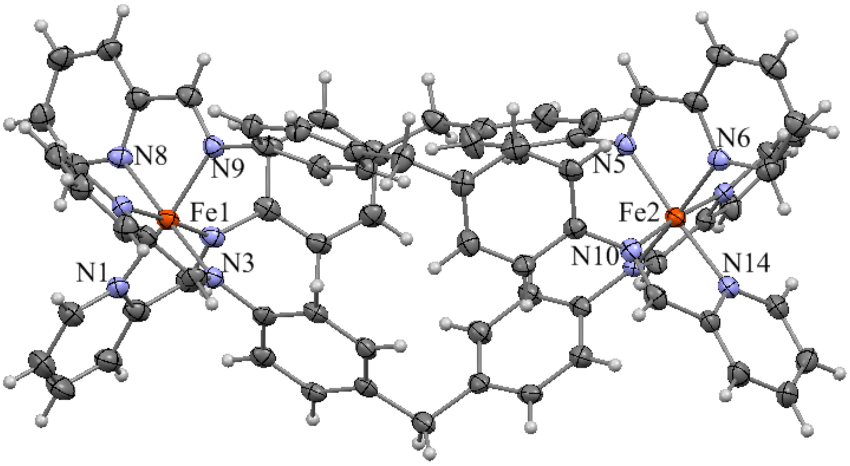

b)

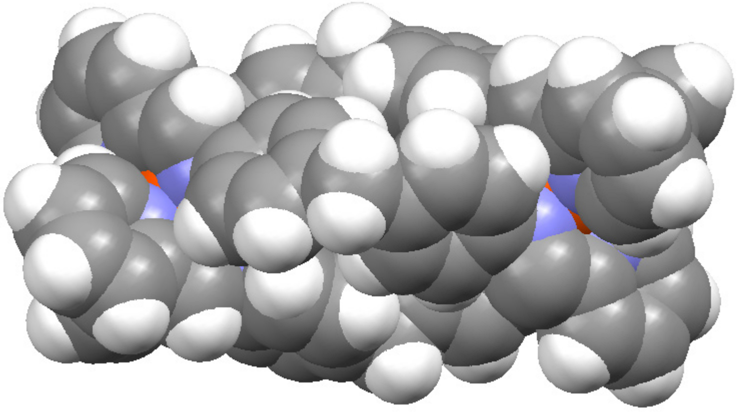

5. X-ray Crystal Data Tables

Table S1. X-Ray Crystal Data Table for 5a

Identification code	5a (CCDC 931620)	
Empirical formula	$C_{42}H_{34}B_2F_8FeN_{12}O_{0.17}$	
Formula weight	938.95	
Temperature	89(2) K	
Wavelength	0.71073 Å	
Crystal system	Trigonal	
Space group	*R*-3	
Unit cell dimensions	*a* = 14.6715(18) Å	*α* = 90°.
	b = 14.6715(18) Å	*β* = 90°.
	c = 37.495(5) Å	*γ* = 120°.
Volume	6989.6(16) Å^3	
Z	6	
Density (calculated)	1.338 Mg/m^3	
Absorption coefficient	0.400 mm^{-1}	
F(000)	2876	
Crystal size	0.38 × 0.30 × 0.16 mm^3	
Theta range for data collection	2.70 to 26.39°	
Index ranges	−18<=h<=18, −18<=k<=18, −46<=l<=46	
Reflections collected	59973	
Independent reflections	3183 [R(int) = 0.0680]	
Completeness to theta = 26.39°	99.7%	
Absorption correction	Semi-empirical from equivalents	
Max. and min. transmission	0.9388 and 0.8628	
Refinement method	Full-matrix least-squares on F^2	
Data/restraints/parameters	3183/0/213	
Goodness-of-fit on F^2	1.084	
Final *R* indices [I>2sigma(I)]	R_1 = 0.0789, wR_2 = 0.2315	
R indices (all data)	R_1 = 0.0881, wR_2 = 0.2382	
Largest diff. peak and hole	1.427 and −0.477 e.Å^{-3}	

In the case of **5a**, a H_2O molecule sitting on a special position was refined isotropically and had its site occupancy fixed at 0.1111. A BF_4^- anion had its site occupancy fixed at 0.6666 in order to balance the charge of the cation.

Table S2. X-Ray Crystal Data Table for 5b.

**Identification code**	**5b (CCDC 931617)**	
Empirical formula	$C_{39}H_{30}B_2F_8FeN_{12}$	
Formula weight	896.22	
Temperature	89(2) K	
Wavelength	0.71073 Å	
Crystal system	Triclinic	
Space group	*P*-1	
Unit cell dimensions	a = 8.7106(6) Å	α = 101.395(4)°.
	b = 13.1870(10) Å	β = 100.194(4)°.
	c = 22.8993(18) Å	γ = 97.669(4)°.
Volume	2498.8(3) Å^3	
Z	2	
Density (calculated)	1.191 Mg/m^3	
Absorption coefficient	0.370 mm^{-1}	
F(000)	912	
Crystal size	0.85 × 0.33 × 0.10 mm^3	
Theta range for data collection	0.93 to 25.50°	
Index ranges	−10<=h<=9, −15<=k<=15, −27<=l<=27	
Reflections collected	37471	
Independent reflections	8971 [R(int) = 0.0715]	
Completeness to theta = 25.50°	96.5%	
Absorption correction	Semi-empirical from equivalents	
Max. and min. transmission	0.7447 and 0.5809	
Refinement method	Full-matrix least-squares on F^2	
Data/restraints/parameters	8971/0/559	
Goodness-of-fit on F^2	1.093	
Final *R* indices [I>2sigma(I)]	R_1 = 0.0945, wR_2 = 0.2581	
R indices (all data)	R_1 = 0.1083, wR_2 = 0.2680	
Largest diff. peak and hole	1.143 and −0.818 e.Å^{-3}	

Following the location of the atoms of **5b** in the ΔF map, there was still residual electron density present within channels through the structure. Disappointingly, this residual electron density could not be satisfactorily modeled. The SQUEEZE routine of PLATON was employed to treat the regions of diffuse solvent. The number of electrons located was 155 per unit cell and this was assigned to a molecule of $CH_3CH_2OCH_2CH_3$ and 3/2 molecules of CH_3CN per complex.

Table S3. X-Ray Crystal Data Table for 6b.

Identification code	6b (CCDC 931619)	
Empirical formula	$C_{68}H_{88}B_4F_{16}Fe_2N_{24}O$	
Formula weight	1716.56	
Temperature	93(2) K	
Wavelength	0.71073 Å	
Crystal system	Monoclinic	
Space group	*P*2(1)/*m*	
Unit cell dimensions	*a* = 13.004(3) Å	*α* = 90°.
	b = 23.348(5) Å	*β* = 92.558(12)°.
	c = 14.674(3) Å	*γ* = 90°.
Volume	4450.8(16) $Å^3$	
Z	2	
Density (calculated)	1.281 Mg/m^3	
Absorption coefficient	0.412 mm^{-1}	
F(000)	1776	
Crystal size	0.54 × 0.41 × 0.15 mm^3	
Theta range for data collection	1.39 to 25.28°	
Index ranges	−15<=h<=15, −27<=k<=27, −17<=l<=17	
Reflections collected	77349	
Independent reflections	8030 [R(int) = 0.2002]	
Completeness to theta = 25.28°	96.9%	
Absorption correction	Semi-empirical from equivalents	
Max. and min. transmission	0.9408 and 0.8081	
Refinement method	Full-matrix least-squares on F^2	
Data/restraints/parameters	8030/0/533	
Goodness-of-fit on F^2	0.852	
Final *R* indices [I>2sigma(I)]	R_1 = 0.0738, wR_2 = 0.2132	
R indices (all data)	R_1 = 0.1023, wR_2 = 0.2336	
Largest diff. peak and hole	1.276 and −1.218 e.$Å^{-3}$	

Similar issues, to those observed for **5b**, were encountered in the case of **6b**. The SQUEEZE routine of PLATON was applied and this located 467 electrons per unit cell. These were assigned to 10 CH_3CN molecules per complex.

Table S4. X-Ray Crystal Data Table for 6c.

**Identification code**	**6c (CCDC 931618)**	
Empirical formula	$C_{63}H_{51}Fe_2N_{27}$	
Formula weight	1298.01	
Temperature	89(2) K	
Wavelength	0.71073 Å	
Crystal system	Monoclinic	
Space group	*C*2/*c*	
Unit cell dimensions	*a* = 41.770(4) Å	*α* = 90°.
	b = 20.297(2) Å	*β* = 95.264(4)°.
	c = 18.751(2) Å	*γ* = 90°.
Volume	15830(3) $Å^3$	
Z	8	
Density (calculated)	1.089 Mg/m^3	
Absorption coefficient	0.418 mm^{-1}	
F(000)	6672	
Crystal size	0.23 × 0.19 × 0.12 mm^3	
Theta range for data collection	0.98 to 24.85°	
Index ranges	−49<=h<=42, −22<=k<=20, −15<=l<=22	
Reflections collected	22939	
Independent reflections	9733 [R(int) = 0.0410]	
Completeness to theta = 24.85°	71.1%	
Absorption correction	Semi-empirical from equivalents	
Max. and min. transmission	0.9729 and 0.6874	
Refinement method	Full-matrix least-squares on F^2	
Data/restraints/parameters	9733/48/829	
Goodness-of-fit on F^2	1.056	
Final *R* indices [I>2sigma(I)]	R_1 = 0.0610, wR_2 = 0.1721	
R indices (all data)	R_1 = 0.0805, wR_2 = 0.1841	
Largest diff. peak and hole	0.748 and −0.346 e.$Å^{-3}$	

The crystals of **6c** were small and weakly diffracting resulting in low completeness (71%). A SIMU restraint was applied to various atoms in the ligand namely C1,C2; C9,C10,C8, N5; C16,C17,C15; N4,C8,N5,C7. Additionally, the SQUEEZE procedure was employed to treat regions of diffuse solvent which could not be sensibly modelled, 2003 electrons per unit cell were located and this was assigned to 4 BF_4 and 4 CH_3CN molecules per complex.

Table S5. X-Ray Crystal Data Table for 6f.

**Identification code**	**6f (CCDC 931616)**	
Empirical formula	$C_{45.50}H_{37.50}B_2F_8FeN_{14.50}$	
Formula weight	1016.87	
Temperature	100(2) K	
Wavelength	0.71080 Å	
Crystal system	Triclinic	
Space group	*P*-1	
Unit cell dimensions	*a* = 15.815(3) Å	*α* = 93.71(3)°.
	b = 16.813(3) Å	*β* = 100.45(3)°.
	c = 18.505(4) Å	*γ* = 93.53(3)°.
Volume	4815.1(17) $Å^3$	
Z	4	
Density (calculated)	1.403 Mg/m^3	
Absorption coefficient	0.394 mm^{-1}	
F(000)	2080	
Crystal size	0.1 × 0.1 × 0.1 mm^3	
Theta range for data collection	1.31 to 26.00°	
Index ranges	−18<=h<=18, −20<=k<=20, −22<=l<=22	
Reflections collected	63141	
Independent reflections	17236 [R(int) = 0.0489]	
Completeness to theta = 26.00°	91.0%	
Absorption correction	None	
Refinement method	Full-matrix least-squares on F^2	
Data/restraints/parameters	17236/18/1284	
Goodness-of-fit on F^2	1.070	
Final *R* indices [I>2sigma(I)]	R_1 = 0.0930, wR_2 = 0.2782	
R indices (all data)	R_1 = 0.1071, wR_2 = 0.2884	
Largest diff. peak and hole	1.092 and −0.661 e.$Å^{-3}$	

In case of structure **6f** CH_3CN carbon atoms C10, C11 and C158 when refined anisotropically had anisotropic displacement parameters (ADPs) uniaxially elongated. An ISOR command was employed to restrain the ADPs of these atoms.

Table S6. X-Ray Crystal Data Table for 8.

**Identification code**	**8 (CCDC 931621)**	
Empirical formula	$C_{77}H_{63}B_4F_{16}Fe_2N_{13}O_{0.54}$	
Formula weight	1637.98	
Temperature	90(2) K	
Wavelength	0.71073 Å	
Crystal system	Monoclinic	
Space group	*P*2(1)/*n*	
Unit cell dimensions	*a* = 22.372(5) Å	*α* = 90°.
	b = 13.462(2) Å	*β* = 107.148(7)°.
	c = 24.944(5) Å	*γ* = 90°.
Volume	7179(3) $Å^3$	
Z	4	
Density (calculated)	1.516 Mg/m^3	
Absorption coefficient	0.503 mm^{-1}	
F(000)	3345.6	
Crystal size	0.67 × 0.30 × 0.07 mm^3	
Theta range for data collection	1.08 to 24.73°	
Index ranges	−26<=h<=25, 0<=k<=15, 0<=l<=29	
Reflections collected	12078	
Independent reflections	12078 [R(int) = 0.0000]	
Completeness to theta = 24.73°	98.5%	
Absorption correction	Semi-empirical from equivalents	
Max. and min. transmission	0.7451 and 0.6945	
Refinement method	Full-matrix least-squares on F^2	
Data/restraints/parameters	12078/0/1075	
Goodness-of-fit on F^2	1.041	
Final *R* indices [I>2sigma(I)]	R_1 = 0.0616, wR_2 = 0.1424	
R indices (all data)	R_1 = 0.1014, wR_2 = 0.1699	
Largest diff. peak and hole	1.350 and −0.529 e.$Å^{-3}$	

In the structure **8** two of the BF_4 ions showed random disorder about a threefold axis. For the anion containing B1 the occupancy of the major component for F1, F3, F4 was 0.52 and for the anion containing B3 the occupancy of the major component for F5, F6, F8 was 0.67. A water molecule was assigned partial site occupancy of 0.54.

6. Overlaid X-ray Crystal Structures

Figure S35. Overlaid X-ray structures of Fe(II) cylinders **6b** (solid lines) and **6c** (dotted lines).

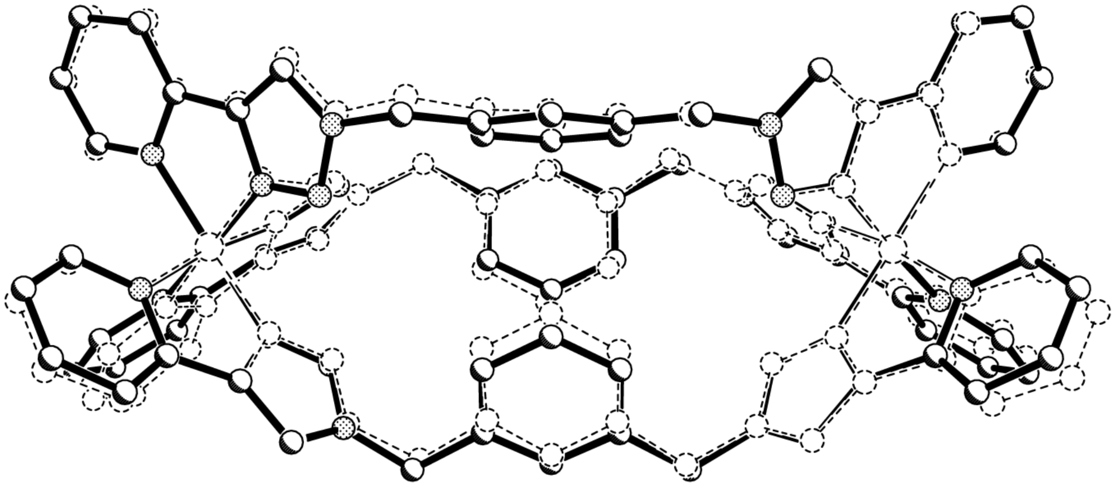

6b	6c	Deviation
N1	N21	0.028
N2	N22	0.067
N5	N1	0.111
N6	N2	0.127
N9	N11	0.139
N10	N12	0.055
Fe1	Fe1	0.035
N1A	N29	0.179
N2A	N28	0.068
N5A	N9	0.013
N6A	N8	0.092
N9A	N19	0.136
N10A	N18	0.148
Fe1A	Fe2	0.038
Weighted R.M.S. deviation:		0.1014 Å

Figure S36. Overlaid X-ray structures of Fe(II) cylinders **1** (solid lines) and **8** (dotted lines).

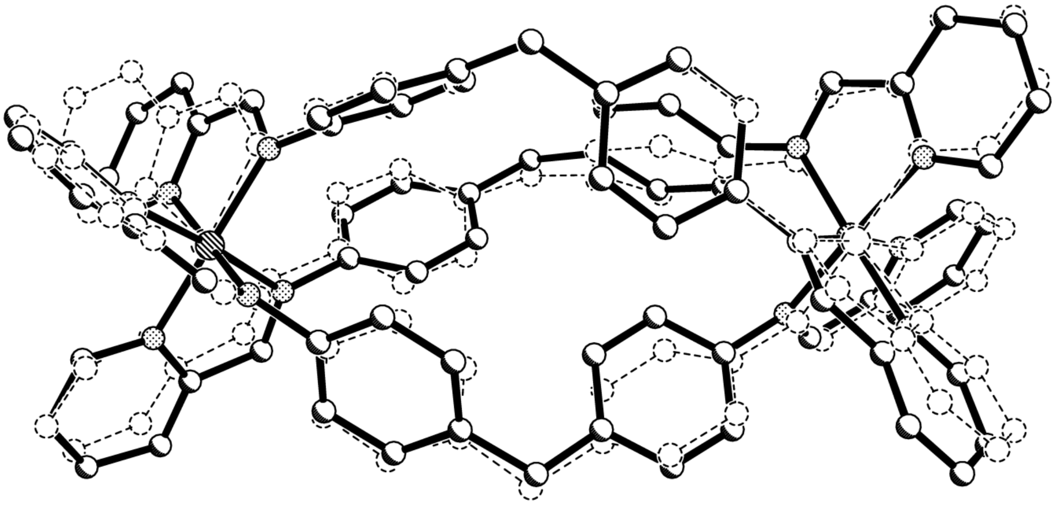

**1**	**8**	**Deviation**
N1	N4	0.312
N3	N3	0.089
N5	N5A	0.412
N6	N6A	0.067
N8	N5	0.263
N9	N6	0.154
N10	N3A	0.291
N12	N2	0.256
N13	N1	0.233
N14	N4A	0.260
N30	N2A	0.235
N31	N1A	0.451
Fe1	Fe1	0.117
Fe2	Fe1A	0.095
Weighted R.M.S. deviation:		0.2572 Å

Figure S37. Overlaid X-ray structures of Fe(II) cylinders **8** (solid lines) and **6f** (dotted lines).

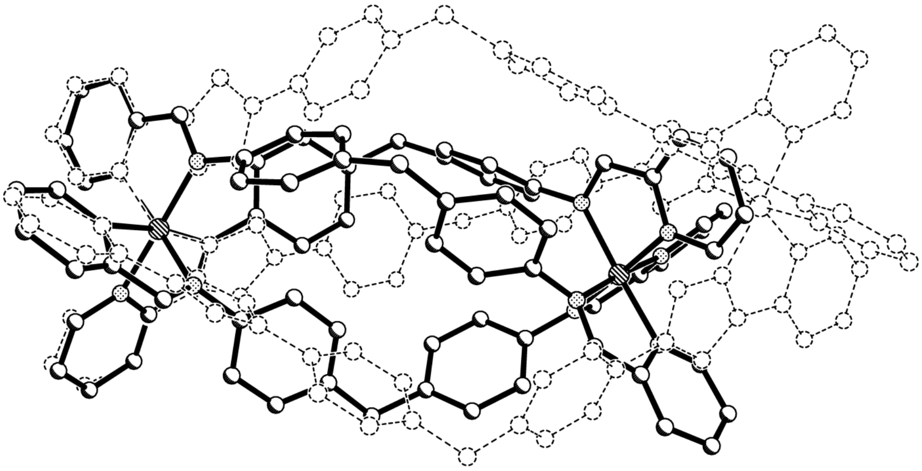

**8**	**6f**	**Deviation**
N1	N6	0.098
N3	N1	0.137
N8	N16	0.155
N9	N16	0.155
N9	N33	0.074
N12	N11	0.188
N13	N5	0.049
Fe1	Fe1	0.052
Weighted R.M.S. deviation:		0.1186 Å

7. Histidine Competition ^{1}H-NMR Experiments

Figure S38. Partial ^{1}H-NMR of a) **3a** in CD_3CN/D_2O (97:3) b) **6a** in CD_3CN/D_2O (97:3) and c) **6a** after the addition of histidine in CD_3CN/D_2O (97:3).

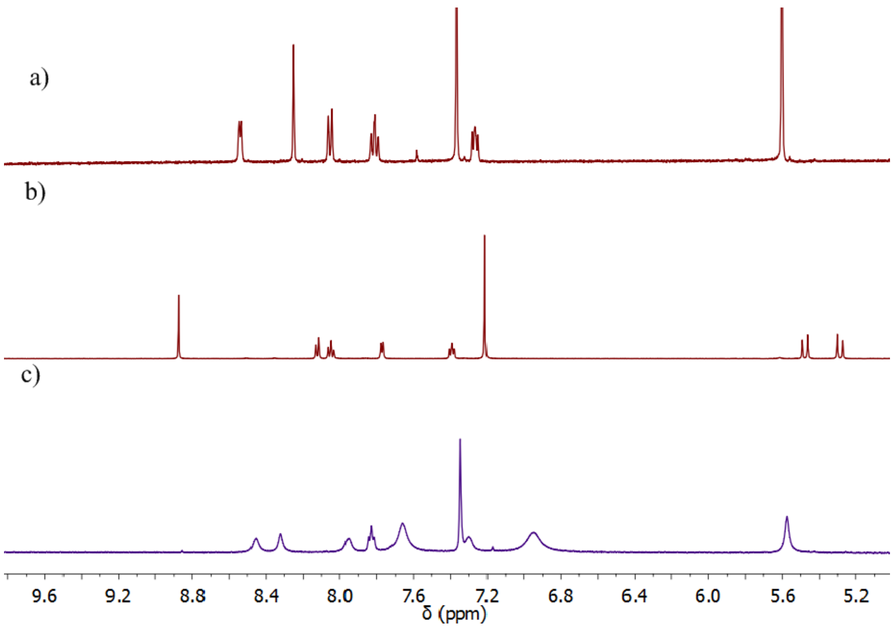

8. UV-Vis Spectra

Figure S39. UV-Vis spectra (CH_3CN, 10^{-5} M) of Fe(II) model complexes **5a** and **5b**.

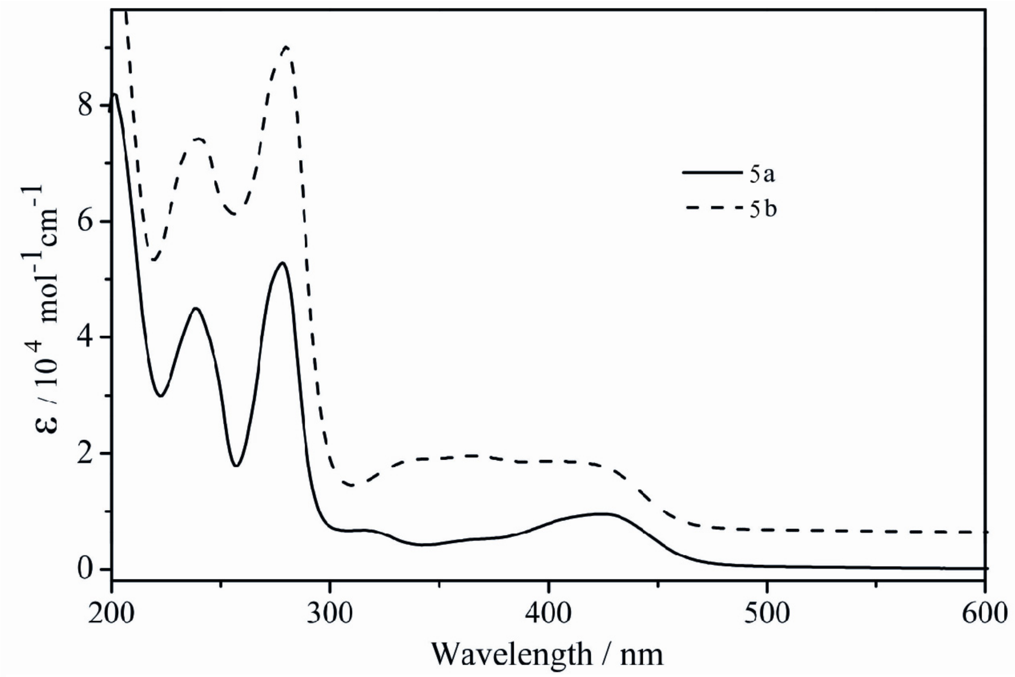

Figure S40. UV-Vis spectra (10^{-4} M) of the Fe(II) cylinder **6a** in acetonitrile and DMSO.

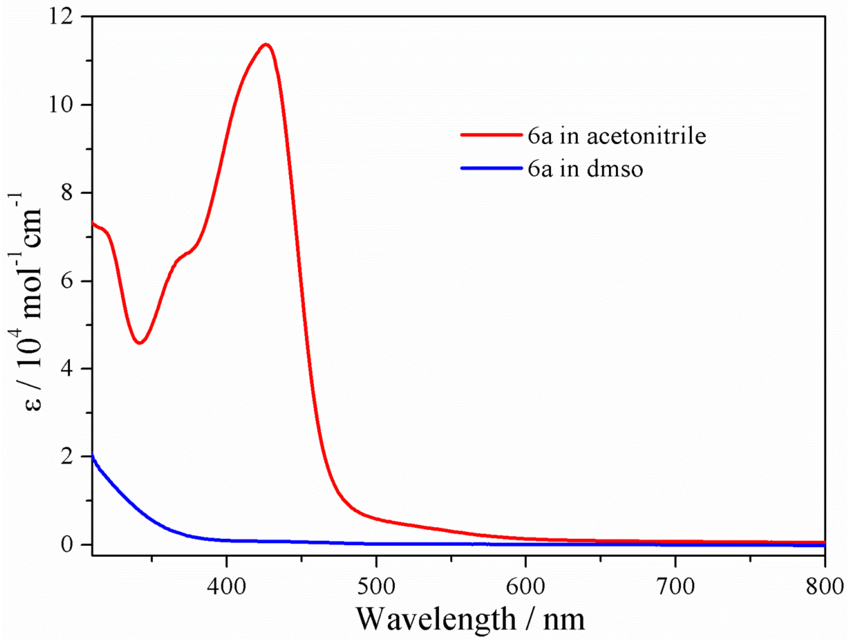

Figure S41. Photograph of the Fe (II) cylinder **6a** obtained at 10^{-4} M concentrations in acetonitrile (left) and DMSO (right).

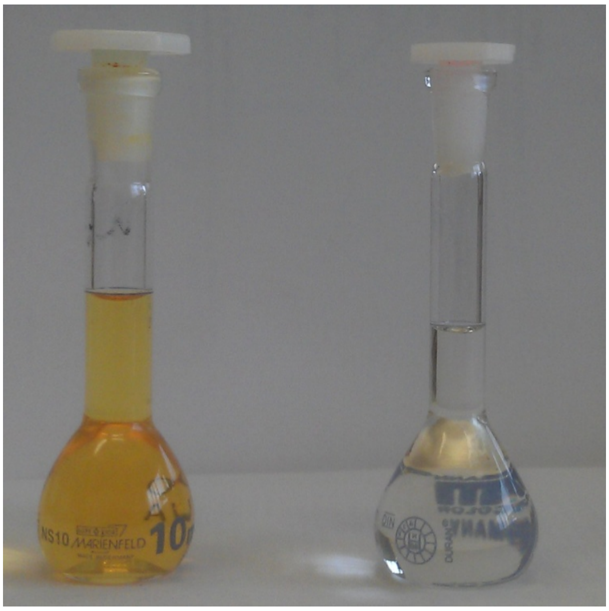

Figure S42. Partial ^{1}H-NMR spectra (500 MHz, d_6-DMSO, 298 K) of a) the ligand **3a** and b) the Fe(II) cylinder **6a.**

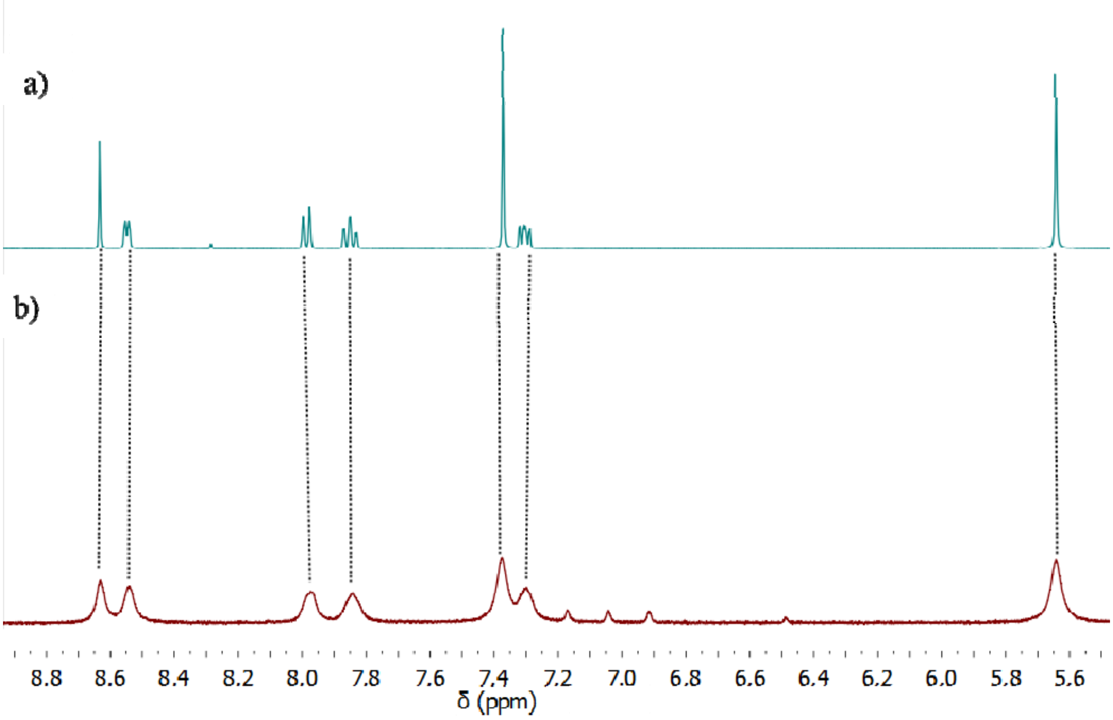

Figure S43. UV-Vis spectra (CH_3CN, 10^{-4} M) of the Fe(II) cylinder **6a** before and after the addition of histidine.

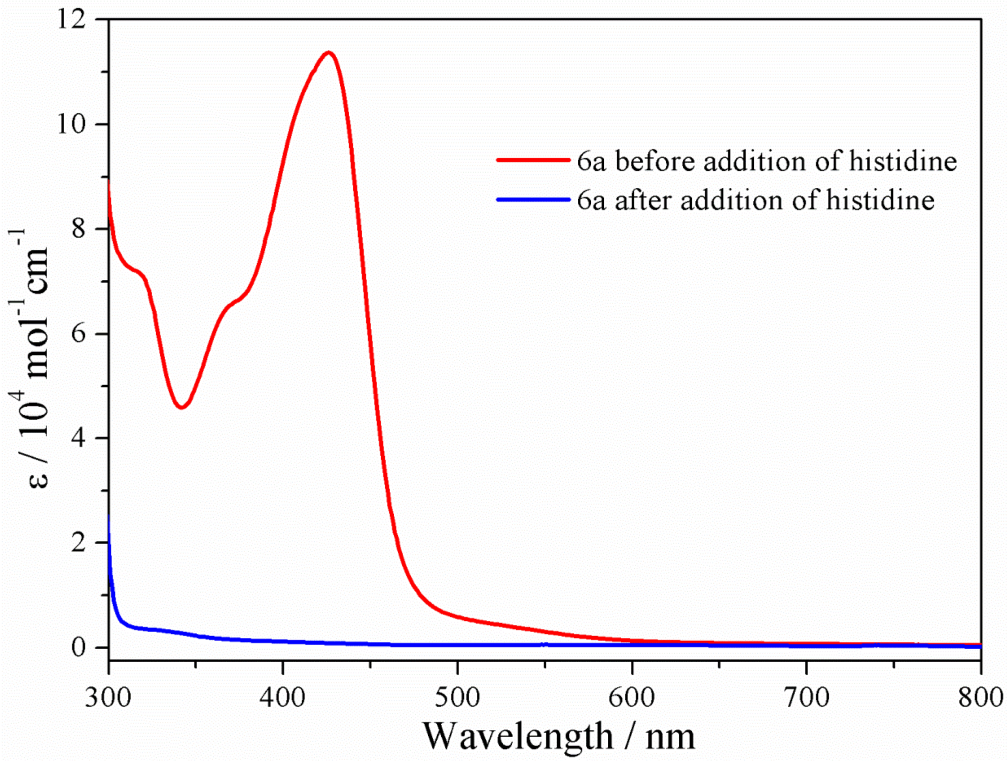

Figure S44. Photograph of the Fe(II) cylinder **6a** obtained at 10^{-4} M concentrations before (left) and after the addition of histidine (right) in acetonitrile.

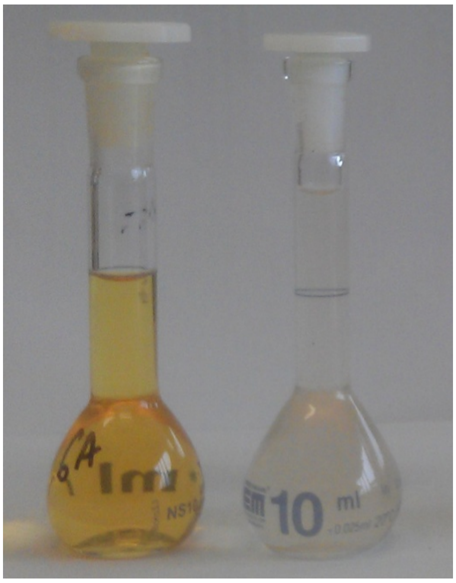

Figure S45. UV-Vis spectra (10^{-4} M) of the Fe(II) cylinder **8** in acetonitrile and DMSO.

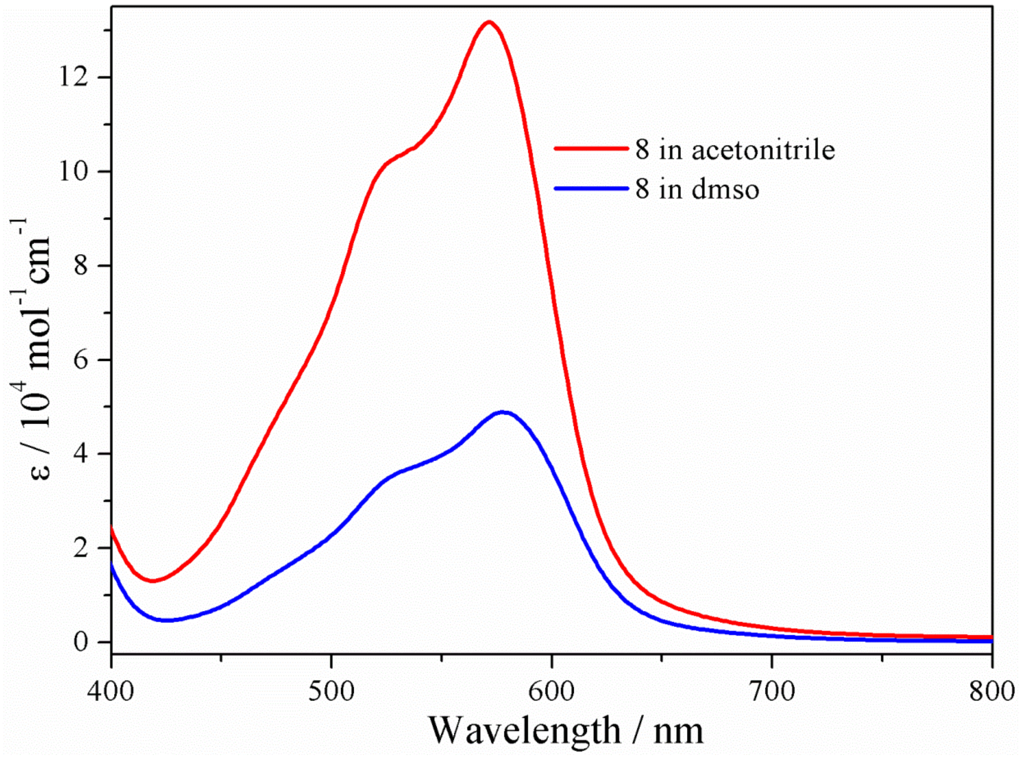

Figure S46. Photograph of the Fe(II) cylinder **8** obtained at 10^{-4} M concentrations in acetonitrile (left) and DMSO (right).

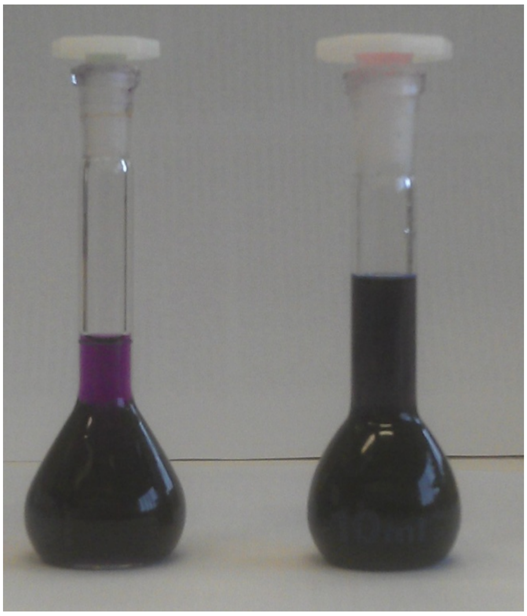

Figure S47. UV-Vis spectra (CH_3CN, 10^{-4} M) of the Fe(II) cylinder **8** before and after the addition of histidine.

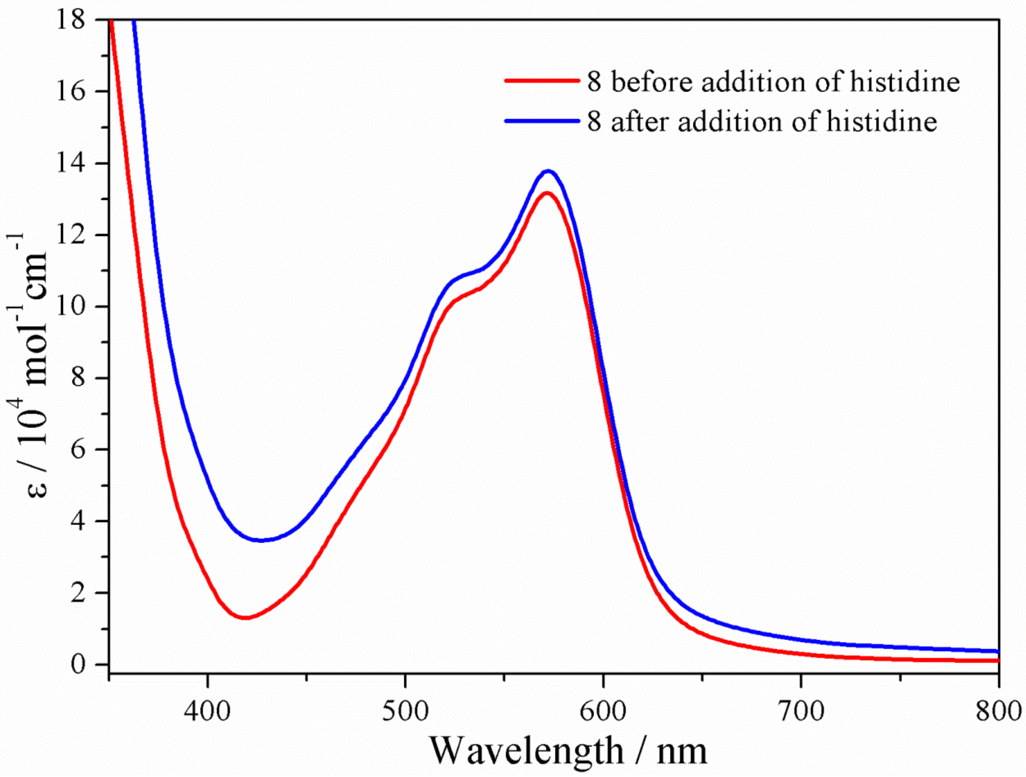

Figure S48. Photograph of the Fe(II) cylinder **8** obtained at 10^{-4} M concentrations before (left) and after (right) the addition of histidine in acetonitrile.

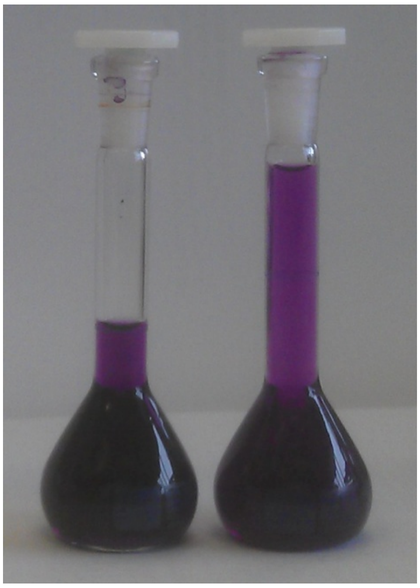

9. Disc Diffusion Assay

Figure S49. Disc diffusion assay showing the effect of DMSO, Amphotericin-B (100 nmol control), the ligands (**3a**–**d**, **4a**, 100 nmol) and the Fe(II) complexes (**5a**, **6a**–**d**, 100 nmol) on *S. cervisiae*.

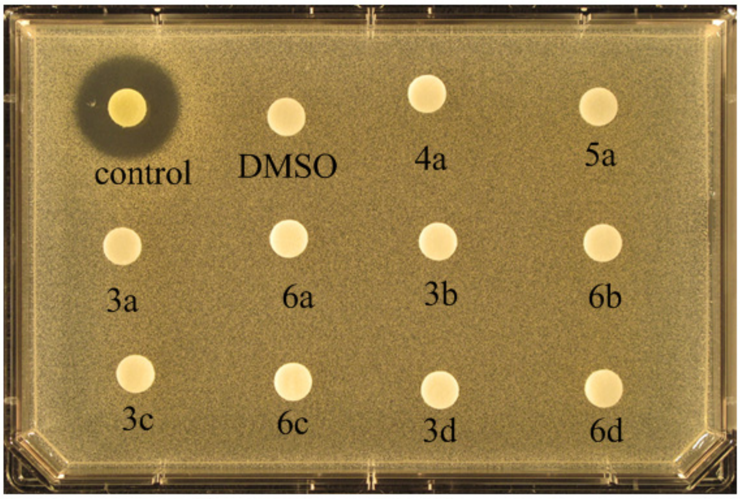

Supplement: Supplementary file 1 [file molecules-18-06383-s001.pdf]
